# Culturally adapted hypocaloric diet improves hepatic steatosis, inflammatory and oxidative biomarkers in Egyptian MASLD patients: a single-arm interventional study

**DOI:** 10.1186/s12944-025-02710-7

**Published:** 2025-09-23

**Authors:** Mohamed Mahmoud Elhoseeny, Fatma Rageh, Nadia Bakry, Rasha Elgamal, Samar S. Ahmed, Samar M. Rezk, Amira A. A. Othman

**Affiliations:** 1 Internal Medicine Department, Faculty of Medicine, Suez University, Suez, Egypt; 2 Infectious Diseases, Gastroenterology and Hepatology Department, Faculty of Medicine, Suez University, Suez, Egypt; 3 Bone Marrow Transplantation and Cord Blood Unit, Mansoura University Children’s Hospital, Mansoura, Egypt; 4 Clinical Pathology Department, Faculty of Medicine, Suez University, Suez, Egypt; 5 Community, Occupational and Environmental Medicine Department, Faculty of Medicine, Suez University, Suez, Egypt; 6 Clinical Nutrition Department, Mahalla Hepatology Teaching Hospital, Gharbyia, El-Mahalla El-Kubra, Egypt

**Keywords:** MASLD, Hypocaloric diet, Oxidative stress, Hepatic steatosis, Egypt, Metabolic dysfunction

## Abstract

**Background:**

Metabolic dysfunction-associated steatotic liver disease (MASLD) represents a growing public health challenge in Egypt, driven by westernized dietary patterns, urbanization, and physical inactivity. Despite lifestyle intervention being the first-line management, data on structured hypocaloric diets tailored to Egyptian patients remain limited, particularly regarding their effects on hepatic steatosis, inflammatory pathways, and oxidative stress biomarkers. This study aimed to evaluate the impact of a culturally adapted 6-month hypocaloric diet on hepatic fat reduction, metabolic parameters, inflammatory-oxidative biomarkers, and lifestyle factors in Egyptian MASLD patients, with additional exploration of weight-independent mechanisms.

**Methods:**

In this single-center interventional trial, 30 newly diagnosed MASLD patients received a personalized hypocaloric diet (500–1000 kcal/day deficit). Outcomes measured at baseline and post-intervention included anthropometrics, liver enzymes, metabolic profile, hepatic steatosis (CAP score), inflammatory markers (TNF-α, MDA), antioxidant enzymes (SOD, CAT), and lifestyle behaviors (physical activity, sleep). Advanced statistical analyses included effect size estimation, multivariate regression, mediation analysis, and subgroup comparisons (lean vs. obese MASLD).

**Results:**

After 6 months, patients achieved significant reductions in weight (− 10.9 kg), BMI (− 3.9 kg/m^2^), and CAP score (− 89.5 dB/m) (all *P* < 0.001). Liver enzymes improved significantly, with ALT decreasing by − 22.2 U/L and AST by − 21.3 U/L (both *P* < 0.001). TNF-α (− 88.2 pg/mL, baseline 166.1 pg/mL) and MDA (− 1.1 nmol/mL, baseline 2.7 nmol/mL) decreased markedly, with large effect sizes (CAP: *d* = 1.9; TNF-α: *d* = 2.1; MDA: *d* = 1.4). Antioxidant biomarkers improved significantly, with SOD increasing by 209% (*d* = 1.8) and CAT by 48.5% (*d* = 1.2) (both *P* < 0.001). Although BMI and weight loss were strongly associated with hepatic fat reduction, TNF-α reduction remained an independent predictor of CAP improvement (β = 0.31, *P* = 0.02), mediating 32% of the diet’s effect after adjusting for BMI. Patients achieving ≥ 5% weight loss were 4.2 times more likely to experience ≥ 10% CAP score reduction. Lean MASLD patients (n = 6) exhibited greater improvements in hepatic fat and inflammation despite less weight loss; however, these findings should be interpreted with caution due to the small subgroup size. Dietary adherence strongly correlated with CAP reduction (*r* = − 0.71, *P* < 0.001) and antioxidant gains.

**Conclusion:**

A culturally tailored hypocaloric diet effectively improved hepatic steatosis, inflammatory status, and antioxidant capacity in Egyptian MASLD patients. These improvements were partially weight-independent and partially mediated by anti-inflammatory responses. These findings support hypocaloric dietary strategies as a potentially scalable therapeutic option for MASLD management in resource-limited settings, though the absence of a control group limits causal inference, and further evaluation of implementation feasibility and cost-effectiveness is warranted. Additional benefits were also observed in lifestyle behaviors such as physical activity and sleep.

## Introduction

Metabolic dysfunction-associated steatotic liver disease (MASLD) is a major global health issue that has recently replaced the term non-alcoholic fatty liver disease (NAFLD) to highlight its metabolic origins [1]. Unlike NAFLD, which is diagnosed mainly by excluding alcohol use, MASLD requires hepatic steatosis in combination with metabolic dysfunction such as obesity, type 2 diabetes, or insulin resistance [2]. This shift reflects recognition that metabolic dysregulation drives disease progression and increases risks of cirrhosis, hepatocellular carcinoma, and cardiovascular mortality.

The terminology for fatty liver disease has evolved considerably. NAFLD was criticized for being vague and exclusion-based, leading to the 2020 proposal of metabolic dysfunction-associated fatty liver disease (MAFLD), which emphasized positive diagnostic criteria and broader clinical relevance [1, 2]. In 2023, major liver associations endorsed MASLD, substituting “steatotic” for “fatty” to improve scientific precision and reduce stigma. MASLD reverts to mutually exclusive subcategories (e.g., MASLD-alcohol, MASLD-viral), restoring etiology-specific subclassification [3]. In this manuscript, we use MASLD terminology, while noting that patients were recruited in 2022 under MAFLD criteria.

MASLD affects nearly one-third of the global population and over 45% of Egyptians, largely driven by Westernized dietary habits and sedentary lifestyles [4, 5]. In Egypt, the “lean MASLD” phenotype—normal BMI with metabolic dysfunction, is especially important yet underexplored [6]. Without intervention, up to 30% of cases progress to metabolic dysfunction-associated steatohepatitis (MASH), underscoring the urgent need for effective, culturally adapted management strategies [7].

Lifestyle modification remains the cornerstone of management, but the optimal dietary model in Egypt is uncertain. Mediterranean and low-carbohydrate diets show benefits in Western settings, yet their adoption is limited in Egypt by cultural dietary habits, economic barriers, and low nutritional awareness [8]. The reliance on carbohydrate-rich staples such as *aish baladi* and *koshari*, coupled with low physical activity and poor sleep quality, may further worsen MASLD through insulin resistance and inflammation [9]. Sleep disruption and sedentary behavior independently amplify hepatic inflammation via circadian misalignment, hormonal disturbance, and reduced energy expenditure [10–13].

Despite the high national burden, interventional research in Egypt remains scarce. No prior clinical trial has evaluated a structured hypocaloric diet addressing not only calorie restriction but also lifestyle factors such as sleep and physical activity. Most regional studies have focused on pharmacological therapy, overlooking scalable non-pharmacological approaches [11]. Additionally, oxidative stress, driven by lipid peroxidation and impaired antioxidant defenses, is a critical but understudied pathway in Egyptian MASLD [12].

Although Mediterranean or low-carbohydrate diets are efficacious elsewhere, their sustainability in Egypt is limited by cost, food availability, and cultural norms [3]. A culturally adapted hypocaloric diet, based on modest energy restriction while preserving traditional foods, may overcome these barriers. Evidence suggests that even moderate caloric deficits (500–750 kcal/day) can improve hepatic steatosis regardless of macronutrient composition [10]. By substituting refined grains with whole grains and increasing legumes, this approach aligns better with local practices and may be particularly relevant for lean MASLD patients.

Therefore, this study evaluated whether a six-month, culturally adapted hypocaloric diet (500–1000 kcal/day deficit) could improve hepatic steatosis, metabolic profile, inflammatory and oxidative stress biomarkers, and lifestyle factors in Egyptian MASLD patients. We hypothesized that the intervention would significantly reduce liver fat and improve biomarkers, with additional benefits in sleep and physical activity.

## Subjects and methods

### Study population and design

This prospective, single-center interventional study was conducted at Mahala Liver Teaching Hospital, Egypt, between May and November 2022. Of 150 consecutive patients screened, 120 were excluded (85 did not meet MASLD criteria [CAP < 248 dB/m or insufficient metabolic risk factors], 25 declined participation, and 10 had comorbid conditions). Thirty participants fulfilling eligibility criteria were enrolled in a 6-month hypocaloric diet intervention.

Recruitment targeted adults aged 18–70 years with newly diagnosed MASLD. Diagnosis was established using transient elastography (FibroScan®, Echosens, France), defined as hepatic steatosis with CAP ≥ 248 dB/m plus at least one metabolic risk factor (BMI ≥ 25 kg/m^2^, type 2 diabetes mellitus [T2DM], or ≥ 2 metabolic abnormalities), following international guidelines [13]. Lean MASLD was defined as BMI < 25 kg/m^2^ with CAP ≥ 248 dB/m and ≥ 1 metabolic abnormality [2, 13].

All participants received the same standardized dietary protocol, with individualized caloric deficits of 500–1000 kcal/day based on baseline total daily energy expenditure (see Nutritional Intervention). Baseline demographics, comorbidities, lifestyle habits, and medications were recorded. To enhance representativeness, recruitment aimed to include both overweight (BMI 25–29.9) and obese (BMI ≥ 30) participants.

As a feasibility, single-arm study, outcomes were assessed pre- and post-intervention to evaluate within-participant changes. While the absence of randomization limits causal inference, this design was chosen due to resource constraints and ethical concerns about withholding a potentially beneficial intervention in a high-risk population without structured dietary care.

Sample size was calculated using Stata/BE v.17, targeting 90% power (α = 0.05, effect size = 0.3) to detect a 20% reduction in CAP score. This threshold was based on prior lifestyle intervention trials, where average CAP reductions ranged from 15–25% [14]. A 5% dropout adjustment was included to account for attrition [15].

### Ethical considerations

Ethical approval was obtained from the Ethics Committee of Suez University, Suez, Egypt (Approval #60,323), and the study adhered to the ethical principles outlined in the Declaration of Helsinki. Written informed consent was obtained from all participants before enrollment. Data confidentiality was maintained by anonymizing patient records, and all data were securely stored following the Egyptian Personal Data Protection Law (Law No. 151/2020).

### Inclusion and exclusion criteria

#### Inclusion criteria

Participants were eligible if they were aged between 18 and 70 years and had a recent diagnosis of metabolic dysfunction-associated steatotic liver disease (MASLD). Diagnosis required the presence of hepatic steatosis, confirmed by transient elastography (FibroScan®) with a controlled attenuation parameter (CAP) score of ≥ 248 dB/m, in addition to at least one indicator of metabolic dysfunction. Metabolic dysfunction was defined according to international guidelines. It included overweight or obesity, type 2 diabetes mellitus, or at least two of the following abnormalities: elevated waist circumference (≥ 102 cm in men or ≥ 88 cm in women), blood pressure ≥ 130/85 mmHg or current antihypertensive treatment, triglyceride levels ≥ 1.70 mmol/L, or prediabetes (fasting glucose 5.6–6.9 mmol/L or 2-h post-load glucose 7.8–11.0 mmol/L). All participants provided written informed consent before enrollment and were required to adhere to study visits and intervention requirements.

#### Exclusion criteria

Exclusion criteria were applied to eliminate confounding factors that could interfere with the metabolic, hepatic, or inflammatory outcomes being measured. Patients with known chronic liver diseases unrelated to metabolic dysfunction, such as hepatitis B virus (HBV), hepatitis C virus (HCV), autoimmune hepatitis, primary biliary cholangitis, or primary sclerosing cholangitis, were excluded due to their distinct pathophysiological mechanisms, which may not respond to dietary intervention. Individuals with decompensated liver disease (Child–Pugh class B or C) or advanced hepatic fibrosis, defined by a liver stiffness measurement greater than 12 kPa, were also excluded, as these conditions are associated with altered hepatic metabolism and systemic inflammation that could confound the study’s outcomes. Alcohol intake exceeding 20 g per day was a further exclusion criterion to avoid overlap with alcohol-related liver disease. Patients were also excluded if they had been hospitalized for medical or surgical conditions in the 30 days before screening, were pregnant, or required nutritional support via enteral or parenteral routes. Additionally, those receiving hepatotoxic or steatogenic medications, including corticosteroids, tamoxifen, methotrexate, amiodarone, or valproate, were excluded. Severe anemia (hemoglobin < 8 g/dL) was also an exclusion criterion, based on WHO guidelines for nutritional intervention safety, as hypocaloric diets may exacerbate nutritional deficiencies in this high-risk subgroup. In addition, recent use (within 3 months) of antibiotics, antioxidant supplements, or anti-inflammatory agents was also considered an exclusion criterion to minimize potential confounding effects on inflammatory and oxidative biomarkers. These agents were excluded because they can acutely influence systemic oxidative and inflammatory markers (e.g., TNF-α, MDA, SOD) independent of dietary change, potentially masking the true biological effect of the intervention. Finally, participants who refused to follow the dietary protocol or were unable to attend follow-up visits were not enrolled.

### Clinical and anthropometric assessment

Weight, height, and body composition (fat mass, muscle mass, total body water [TBW]) were measured using a bioelectrical impedance analyzer (InBody 270, Korea). Although actual water intake was not tracked through dietary records, TBW was used as an indirect indicator of hydration and body composition status pre- and post-intervention. All assessments were conducted in the morning after ≥ 12 h of fasting and abstention from physical activity to minimize short-term hydration effects on TBW estimates. BMI was calculated as weight (kg) divided by height (m^2^), with measurements taken in light clothing and without shoes to minimize variability. Waist and hip circumferences were measured in triplicate using a non-stretch tape, and the waist-to-hip ratio (WHR) was calculated as an indicator of visceral adiposity [16].

Sociodemographic and lifestyle data, including age, sex, education level, occupation, and smoking status, were collected using structured questionnaires. Smoking was classified as never (no history), past (ceased ≥ 6 months prior), or current (actively smoking at enrollment). Associations between smoking status and CAP score, TNF-α, MDA, SOD, and CAT were evaluated. Blood pressure was measured using an automated sphygmomanometer (Omron HEM-7120, Japan), following standard protocols.

### Laboratory investigations

Fasting venous blood samples were collected in the morning (8:00–10:00 AM) after a 12-h fast. Plasma was separated by centrifugation at 3,000 rpm for 15 min and stored at − 80 °C until analysis. Liver function tests, including alanine aminotransferase (ALT) and aspartate aminotransferase (AST), as well as lipid profile (total cholesterol, low-density lipoprotein [LDL], high-density lipoprotein [HDL], and triglycerides), and fasting glucose were analyzed using an automated Cobas c 501 analyzer (Roche Diagnostics, Switzerland) [17].

Inflammatory biomarkers, including tumor necrosis factor-alpha (TNF-α) and malondialdehyde (MDA), were quantified using enzyme-linked immunosorbent assay (ELISA) kits (R&D Systems, USA). Antioxidant enzyme activities, including superoxide dismutase (SOD) and catalase (CAT), were assessed via spectrophotometric assays using commercially available kits (Cayman Chemical, USA). These markers were chosen due to their established roles in MASLD pathogenesis and response to dietary intervention [18].

All ELISA assays were performed in duplicate, following manufacturer's protocols. Each run included calibration standards, blanks, and internal controls to ensure reliability. The intra-assay coefficients of variation (CVs) were 5.2% for TNF-α, 4.8% for MDA, 6.1% for SOD, and 7.3% for CAT, with inter-assay CVs < 10% across all analytes.

Biochemical analyses, including liver enzyme and lipid profile measurements, were conducted in an ISO 15189-accredited laboratory to ensure high precision and reliability, critical for accurate assessment of hepatic steatosis and insulin resistance in MASLD patients.

### Nutritional intervention

Participants were prescribed a culturally adapted hypocaloric diet providing a 500–1000 kcal/day deficit from their baseline intake of 2200 ± 300 kcal/day, as assessed by a validated Egyptian Food Frequency Questionnaire [19]. Energy requirements were estimated using the Mifflin–St. Jeor equation with individualized adjustments for physical activity, and total daily energy expenditure (TDEE) was also calculated using the Harris–Benedict equation with activity factors (sedentary = 1.2, moderate = 1.55) guided by the Egyptian-validated Physical Activity Questionnaire (PAQ). The planned deficit aimed for gradual weight loss (~ 0.5–1 kg/week) while ensuring nutritional adequacy by never setting total energy intake below 1200 kcal/day. The intervention emphasized culturally appropriate, MASLD-targeted foods including whole grains (≥ 3 servings/day of brown rice or whole wheat bread), lean proteins (1.2–1.5 g/kg/day from fish, poultry and legumes), and polyunsaturated fats (20–30 g/day from sunflower oil and nuts), and it restricted refined carbohydrates (< 50 g/day), sugar-sweetened beverages (≤ 1/week), and fried foods (≤ 1/week). Macronutrient targets were defined to reflect Mediterranean-style principles adapted for Egyptian populations, with an overarching goal of approximately 40% carbohydrates, 30% protein and 30% fat (< 7% saturated); plans were individualized and in practice allowed tailoring (approximately 40–50% carbohydrates and 20–30% protein) to accommodate cultural preferences while preserving the higher protein target chosen to enhance satiety, preserve lean mass, and improve insulin sensitivity in participants with obesity (mean BMI 38.6 kg/m^2^; Table 1). Dietary sources emphasized whole grains, legumes, lean meats, fish, low-fat dairy, vegetables, and fruits while minimizing refined sugars and saturated fats; micronutrient adequacy was supported by iron-rich foods (legumes, poultry, eggs), folate-containing vegetables (leafy greens, legumes), and vitamin B12 sources (fish, dairy, eggs). No iron supplementation was prescribed so that any hematologic stability or improvement could be attributed to dietary intake rather than pharmacologic correction [20, 21].

Adherence was quantified monthly using 3-day food records (two weekdays and one weekend day), supplemented by weekly 24-h recalls and FFQ data; records were reviewed by a registered dietitian. A composite adherence score (0–100%) combined caloric compliance (40%), macronutrient targets (30%) and food-group frequency (30%): Adherence Score (%) = Caloric Compliance (40%) + Macronutrient Compliance (30%) + Food Group Compliance (30%), where caloric compliance reflected the percentage of recorded days within the prescribed 500–1000 kcal/day deficit, macronutrient compliance captured alignment with target distribution (carbohydrates 40–50%, protein 20–30%, fat ≤ 30%), and food-group compliance assessed the frequency of meeting goals for whole grains, lean protein, vegetables and healthy fats. The score’s validity is supported by prior correlations with objective biomarkers (e.g., plasma carotenoids, r = 0.62) in Egyptian cohorts, and the ≥ 80% cutoff aligns with thresholds used in Mediterranean diet trials for MASLD; participants scoring ≥ 80% were classified as adherent [14, 19], and 85% of participants in this cohort maintained > 80% compliance throughout the 6-month intervention. Minimum caloric intake was set at 1,200 kcal/day for women and 1,500 kcal/day for men to ensure nutritional adequacy. This nutritional approach was designed to simultaneously address hepatic steatosis while accommodating local dietary preferences and availability [22].

### Lifestyle assessment

Physical activity was assessed using the Egyptian-validated Physical Activity Questionnaire (PAQ), which categorizes individuals as sedentary (< 3 MET-hours/week), moderately active (3–6 MET-hours/week), or highly active (> 6 MET-hours/week) [23]. Sleep quality was evaluated using the Arabic-translated Athens Insomnia Scale (AIS) (scores ≥ 6 indicating clinically significant disturbances) [24]. Activity/sleep were stabilized using accelerometry (ActiGraph GT3X +). Participants with > 10% deviation in daily steps/sleep duration were excluded to minimize confounding.

Symptom improvement (energy, sleep quality, cognition, and mood) was tracked using structured interviews and a 5-point Likert scale, administered at baseline and post-intervention. Psychological status was assessed via structured interviews using a 5-point Likert scale (1 = severe impairment, 5 = no impairment) for mood and cognitive symptoms. Psychological improvement was defined as: (1) ≥ 1-point increase on the 5-point Likert scale for mood AND (2) patient-reported enhancement in ≥ 1 of: stress tolerance, cognitive clarity, or emotional stability during follow-up interviews. This scale was adapted from prior behavioral and lifestyle intervention studies, adapted from the validated Profile of Mood States (POMS) questionnaire [25], and pre-tested for clarity during participant onboarding to ensure consistent interpretation.

### Hepatic steatosis and fibrosis assessment

Hepatic steatosis and fibrosis were assessed using FibroScan®. Steatosis severity was classified by CAP score as mild (248–260 dB/m), moderate (260–275 dB/m), or severe (> 275 dB/m) [13]. These thresholds are consistent with the meta-analysis by Karlas et al. (2017), which proposed optimal cut-offs of 248, 268, and 280 dB/m for histologically confirmed S1, S2, and S3 steatosis, respectively, in a global cohort [13]. Our chosen cut-offs fall within the reported 95% confidence intervals for S2 (257–284 dB/m) and S3 (268–294 dB/m), and were adapted to our Egyptian MASLD population, characterized by high obesity prevalence (mean BMI 38.6 kg/m^2^), severe steatosis (66.7%), and metabolic dysfunction (36.7% with diabetes) (Table 1). We acknowledge that other thresholds, such as those reported by Lee et al. (2016) (247, 280, 300 dB/m), differ according to ethnicity, reference modality (MRS), and diagnostic criteria [26]. To define a clinically meaningful response, we applied a ≥ 10% reduction in CAP score, consistent with prior lifestyle intervention trials reporting average CAP reductions of 15–25% in MASLD populations. Although no Egyptian-specific CAP validation studies are currently available, the consistency of our CAP findings with ultrasound-based steatosis grading supports the internal validity of our results. Future research should aim to establish locally validated CAP thresholds that incorporate ethnic and dietary factors unique to Egyptian and regional MASLD cohorts.

Liver stiffness measurement (LSM) was used to assess fibrosis, categorized from F0 (no fibrosis) to F4 (cirrhosis). All measurements were performed by a trained hepatologist, with median values derived from at least ten valid readings [25]. Operators were trained and certified, intra-observer variability was minimized by standardized procedures, and both FibroScan® assessments and biomarker analyses were conducted by assessors blinded to timepoint (baseline vs. post-intervention) and dietary adherence, thereby reducing detection bias.

### Outcome measures

The primary outcome was the change in CAP score post-intervention. Secondary outcomes included changes in liver enzymes (ALT, AST), metabolic parameters (BMI, lipid profile, fasting glucose), inflammatory markers (TNF-α, MDA), antioxidant biomarkers (SOD, CAT), and lifestyle factors (physical activity, sleep quality).

CAP was selected as the primary outcome because it provides a validated, non-invasive, and quantitative assessment of hepatic steatosis, the hallmark of MASLD. Unlike liver enzymes or inflammatory markers, CAP directly reflects intrahepatic fat burden and is sensitive to changes induced by dietary modification. Its clinical utility is endorsed by the 2021 EASL guidelines for non-invasive liver assessment.

### Qualitative analysis

Qualitative feedback was obtained via open-ended interview questions at study end, asking participants about their experiences, challenges, and perceived benefits. An inductive thematic analysis was conducted. Two investigators independently reviewed the responses, coded the data manually, and generated themes through consensus. Discrepancies were resolved through discussion. Representative quotes were selected to illustrate key themes.

### Statistical analysis

Data analysis was performed using SPSS v.27 (IBM, USA), and data visualization was performed using additional tools such as Microsoft Excel and Python to supplement SPSS-based analyses. No missing data required imputation as all 30 participants completed the study protocol with full datasets. Continuous variables were tested for normality using the Shapiro–Wilk test. Normally distributed data were expressed as mean ± standard deviation (SD) and analyzed using paired t-tests for pre- and post-intervention comparisons. For multiple comparisons (e.g., subgroup analyses of lean vs obese MASLD), we applied Bonferroni correction, adjusting the significance threshold to P < 0.025 for these specific analyses.

Continuous variables were presented as mean ± standard deviation and compared using paired t-tests for pre- and post-intervention changes. Categorical variables measured at baseline and 6 months in the same individuals (e.g., physical activity level, sleep quality, symptom improvement) were analyzed using the McNemar test. A *P*-value < 0.05 was considered statistically significant for primary outcomes, while secondary outcomes were interpreted with consideration of effect sizes. Effect sizes (Cohen's *d* for t-tests, r for correlations) were calculated to assess the magnitude of observed changes.

Non-normally distributed variables were presented as median (interquartile range) and analyzed using the Wilcoxon signed-rank test. Categorical variables were compared using the chi-square test.

Multivariate linear regression analysis was conducted to identify independent predictors of CAP score reduction, adjusting for age, sex, baseline BMI, and inflammatory biomarkers. Model assumptions were verified through residual analysis and variance inflation factors (< 5 for all predictors). Multicollinearity was assessed using Variance Inflation Factors (VIF), and all predictors had VIF < 2.1. Model fit was reported using adjusted R^2^ for linear regression and Nagelkerke R^2^ for logistic regression to reflect the proportion of variance explained.

Spearman’s correlation was used to explore associations between changes in hepatic steatosis and oxidative stress markers. Binary logistic regression was used to determine predictors of achieving a ≥ 10% reduction in CAP score. Mediation analysis was conducted to explore whether reductions in TNF-α or BMI mediated the effect of the intervention on hepatic fat content. For clinical interpretability, CAP scores were also categorized into mild (248–260 dB/m), moderate (260–275 dB/m), and severe (> 275 dB/m) steatosis to evaluate categorical improvement. Statistical significance was set at *P* < 0.05. For mediation analysis, we used bootstrapping with 5,000 resamples to generate 95% confidence intervals, which provides robust estimates without requiring normality assumptions.

Subgroup analysis was performed to compare outcomes between lean MASLD patients (defined as BMI < 25 kg/m^2^) and obese MASLD patients (defined as BMI ≥ 30 kg/m^2^), according to established criteria, aiming to explore potential differences in hepatic steatosis and inflammatory response based on adiposity phenotype, as well as between anaemic and non-anaemic patients at baseline, using ANOVA or independent t-tests for normally distributed continuous outcomes and Mann–Whitney U tests for non-normal data.

Additionally, a dietary adherence score (range 0–100%) was calculated based on weekly 24-h dietary recalls and Food Frequency Questionnaires (FFQ), and its correlation with key clinical outcomes was assessed using Spearman’s correlation analysis. Effect size analysis (Cohen’s d) was applied for all major outcomes to interpret the magnitude of changes.

## Results

### Patient characteristics at baseline: high prevalence of severe steatosis and metabolic comorbidities

This study included 30 newly diagnosed Egyptian patients with MASLD (Table 1). The cohort was relatively young (mean age 39.9 ± 11.9 years) with equal gender distribution (15 males, 15 females), reflecting early disease onset in both sexes. Two-thirds lived in urban areas and were employed, consistent with high exposure to processed, carbohydrate-rich diets and low physical activity, factors that likely contribute to disease development. Educational attainment was low, with 80% able only to read and write, underscoring the need for interventions tailored to populations with limited health literacy.

**Table 1:** Baseline demographic and clinical characteristics of the study population (*n* = 30)

Characteristic	Total (*n* = 30)
**Age (years)**	39.9 ± 11.9
**Sex, n (%)**	
**-** Male	15 (50%)
**-** Female	15 (50%)
**Marital Status, n (%)**	
- Single	4 (13.3%)
- Married	23 (76.7%)
- Divorced	3 (10%)
- Widow	0 (0%)
**Education, n (%)**	
- Highly educated	6 (20%)
- Read and write	24 (80%)
**Occupation, n (%)**	
- Employed	20 (66.7%)
- Unemployed	10 (33.3%)
**Residency, n (%)**	
- Urban	20 (66.7%)
- Rural	10 (33.3%)
**BMI (kg/m**^**2**^**)**	38.6 ± 3.9
**Steatosis Severity, n (%)**	
- Mild (S1: 248–260 dB/m)	3 (10%)
- Moderate (S2: 260–275)	7 (23.3%)
- Severe (S3: > 275)	20 (66.7%)
**Smoking Status, n (%)**	
- Never	18 (60%)
**-** Past	7 (23%)
- Current	5 (17%)
**Comorbidities, n (%)**	
- Diabetes mellitus	11 (36.7%)
- Hypertension	6 (20%)
- Anemia	10 (33.3%)
- Hyperlipidemia	3 (10%)
- Cardiac disease	1 (3.3%)

*BMI* Body Mass Index, *CAP* Controlled Attenuation Parameter, *SD* Standard Deviation

At baseline, 66.7% of patients had severe hepatic steatosis (S3; CAP > 275 dB/m), suggesting delayed diagnosis until advanced stages, which raises concern for progression to fibrosis or cirrhosis without intervention. Comorbidities were frequent: 36.7% had diabetes, 20% hypertension, and 33.3% anemia, each of which worsens metabolic liver disease outcomes. Smoking distribution was 60% never, 23% past, and 17% current. While current smokers had slightly higher TNF-α levels (170 ± 10 pg/mL) than never smokers (162 ± 8 pg/mL; *P* = 0.06), this difference was not significant, and no differences in baseline CAP scores or post-intervention CAP reductions were observed by smoking status.

Anemia, defined as hemoglobin < 12 g/dL in women or < 13 g/dL in men, was present in 10 patients (33.3%). Post-hoc subgroup analysis showed no significant differences between anemic and non-anemic patients in CAP reduction (–60 ± 16 vs. –63 ± 14 dB/m, *P* = 0.48) or TNF-α change (–21 ± 7 vs. –24 ± 5 pg/mL, *P* = 0.32), indicating that anemia did not significantly influence response to the hypocaloric diet.

These findings highlight a metabolically vulnerable cohort with high rates of obesity, diabetes, and advanced steatosis, combined with socioeconomic challenges such as low literacy and urban residency. Such factors must be considered when designing accessible and sustainable lifestyle interventions for MASLD.

### Dietary patterns and nutritional habits at baseline among Egyptian MASLD patients

Baseline dietary intake revealed a strong dependence on refined carbohydrates and limited dietary diversity (Table 2). Most participants reported frequent consumption of staple carbohydrate sources, with rice or macaroni forming a daily component of the diet in over four-fifths of the cohort, while balady bread remained the dominant bread consumed. White bread was less common, typically eaten on a weekly basis. Vegetable consumption was generally adequate, with fresh vegetables included daily by the majority, although fruit intake was markedly lower, as only about one-third reported daily use. Protein sources were skewed toward dairy and poultry, which were consumed regularly, whereas fish and seafood were almost absent from the diet. Eggs were a routine component of meals, either daily or weekly, and legumes were also common. Lipid intake was dominated by daily use of vegetable oils, while hydrogenated oils and butter appeared much less frequently. Processed foods and sugar-sweetened beverages were present but not consumed daily, with canned juices universally taken on a monthly basis and carbonated drinks used by most participants at least occasionally.

**Table 2:** Baseline dietary habits and food frequency in the study population (*n* = 30)

Food Pattern	Daily	Weekly	Monthly	Yearly
**Vegetables**				
- Cooked vegetables	25 (83.3%)	5 (16.7%)	0 (0%)	0 (0%)
- Fresh vegetables	26 (86.7%)	4 (13.3%)	0 (0%)	0 (0%)
**Fruits**				
- Fresh fruits	9 (30%)	21 (70%)	0 (0%)	0 (0%)
- Canned juices	0 (0%)	0 (0%)	30 (100%)	0 (0%)
**Drinks**				
- Carbonated drinks	2 (6.7%)	10 (33.3%)	18 (60%)	0 (0%)
- Stimulant drinks	25 (83.3%)	5 (16.7%)	0 (0%)	0 (0%)
**Processed & Junk Foods**				
- Processed foods	0 (0%)	0 (0%)	7 (23.3%)	23 (76.7%)
- Junk foods	0 (0%)	0 (0%)	3 (10%)	27 (90%)
**Bread**				
- Balady bread	24 (80%)	6 (20%)	0 (0%)	0 (0%)
- White bread	7 (23.3%)	21 (70%)	2 (6.7%)	0 (0%)
**Carbohydrates**				
- Rice or macaroni	25 (83.3%)	5 (16.7%)	0 (0%)	0 (0%)
- Whole grains	28 (93.3%)	2 (6.7%)	0 (0%)	0 (0%)
**Legumes**	16 (53.3%)	13 (43.3%)	1 (3.3%)	0 (0%)
**Protein**				
- Milk & products	24 (80%)	6 (20%)	0 (0%)	0 (0%)
- Meat & poultry	9 (30%)	21 (70%)	0 (0%)	0 (0%)
- Fish & seafoods	0 (0%)	27 (90%)	3 (10%)	0 (0%)
- Eggs	10 (33.3%)	20 (66.7%)	0 (0%)	0 (0%)
**Lipid**				
- Vegetable oils (poly)	30 (100%)	0 (0%)	0 (0%)	0 (0%)
- Hydrogenated oils	3 (10%)	8 (26.7%)	8 (26.7%)	11 (36.7%)
- Butter	2 (6.7%)	1 (3.3%)	0 (0%)	27 (90%)
- Vegetable oils (mono)	0 (0%)	0 (0%)	4 (13.3%)	26 (86.7%)

Data are presented as frequency and percentage of patients reporting consumption at the specified frequency. Abbreviations: n, number of patients

These baseline dietary features reflect a pattern of high glycemic load, driven by reliance on refined grains such as rice, macaroni, and white bread, combined with low intake of antioxidant-rich foods, particularly fruits and fish. Such imbalances are consistent with the elevated baseline levels of inflammatory and oxidative stress markers observed in this cohort (TNF-α 166.1 pg/mL; MDA 2.7 nmol/mL). Although fructose intake was not quantified directly, monthly canned juice consumption and the regular intake of carbonated beverages suggest intermittent fructose exposure, which may have promoted hepatic de novo lipogenesis and influenced triglyceride metabolism. Importantly, after the intervention, both TNF-α (–53.2%) and MDA (–40.7%) improved substantially, indicating that beyond caloric restriction, the dietary shift toward whole grains, legumes, lean proteins, and polyunsaturated fats contributed mechanistically to metabolic recovery.

### Hypocaloric diet improves body composition and anthropometric markers in MASLD patients

**Fig. 1:**
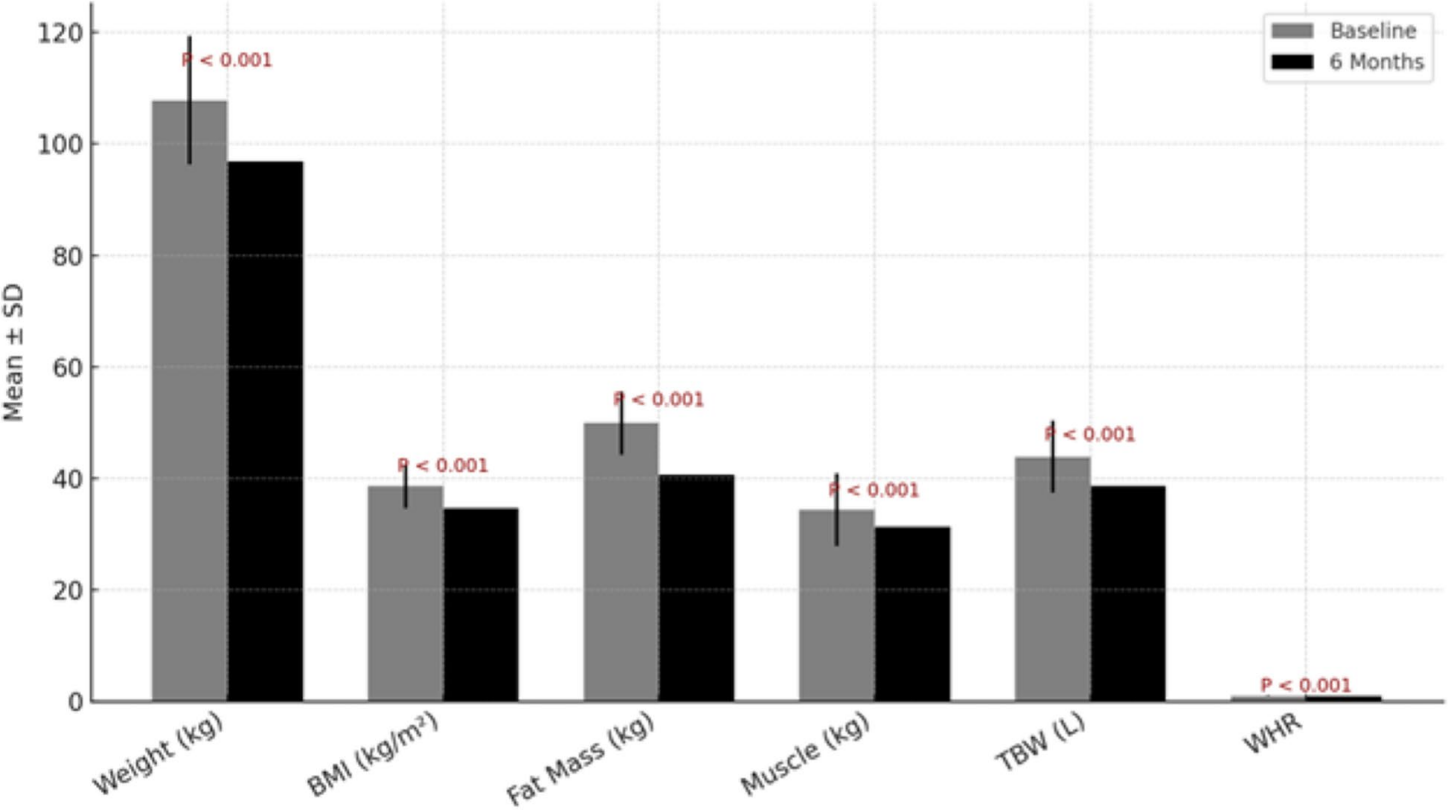
Changes in anthropometric and body composition parameters after 6-month hypocaloric diet in Egyptian MASLD patients (*n* = 30). Bar chart comparing baseline and 6-month measurements for weight, BMI, fat mass, muscle mass, total body water (TBW), and waist-to-hip ratio (WHR). All changes were statistically significant (*P* < 0.001) and are shown as mean ± standard deviation

The 6-month hypocaloric diet produced significant improvements across all anthropometric and body composition parameters (Table 3, Fig. 1). Mean weight loss was 10.9 kg (− 10.1%, *P* < 0.001), exceeding the 7% threshold typically associated with improvements in hepatic steatosis and potential reversal of early fibrosis. BMI declined by 3.9 kg/m^2^, consistent with substantial mass reduction in a cohort with an average baseline BMI of 38.6 ± 4.2 kg/m^2^, spanning different classes of obesity.

**Table 3:** Changes in anthropometric and body composition parameters after 6-month hypocaloric diet in Egyptian MASLD Patients (*n* = 30)

Parameter	Baseline	6 Months	% Change	Mean Difference (95% CI)	Effect Size (d)	*P*-value
Weight (kg)	107.8 ± 11.5	96.9 ± 11.0	− 10.1%	− 10.9 (− 12.1 to − 9.7)	1.8	< 0.001
BMI (kg/m^2^)	38.6 ± 3.9	34.7 ± 3.8	− 10.1%	− 3.9 (− 4.3 to − 3.5)	1.7	< 0.001
Fat mass (kg)	49.9 ± 5.7	40.7 ± 5.6	− 18.4%	− 9.2 (− 10.1 to − 8.3)	1.6	< 0.001
Muscle (kg)	34.4 ± 6.5	31.3 ± 6.9	− 9.0%	− 3.1 (− 3.7 to − 2.5)	1.0	< 0.001
TBW (L)	43.9 ± 6.5	38.7 ± 5.4	− 11.8%	− 5.2 (− 6.3 to − 4.1)	1.1	< 0.001
WHR	1.01 ± 0.04	0.97 ± 0.05	− 4.0%	− 0.04 (− 0.05 to − 0.03)	1.2	< 0.001

Data presented as mean ± standard deviation. % Change = ((Post − Pre)/Pre) × 100. Mean difference represents the change from baseline to 6 months with the corresponding 95% confidence interval (CI). Effect size (Cohen’s d) was calculated to assess the magnitude of change. Abbreviations: *BMI *Body Mass Index, *TBW *Total Body Water, *WHR *Waist-to-Hip Ratio

Body composition analysis showed that weight loss was primarily attributable to adipose tissue reduction, with fat mass decreasing by 9.2 kg (effect size d = 1.6). Muscle mass also declined by 3.1 kg (− 9.0%, *P* < 0.001), a predictable finding during caloric restriction, though clinically relevant given the association of sarcopenia with MASLD progression. Total body water fell by 5.2 L, reflecting reductions in extracellular fluid.

Central adiposity improved, as shown by a 0.04-unit reduction in waist-to-hip ratio (*P* < 0.001). Overall, the intervention achieved meaningful reductions in weight, fat mass, lean mass, and regional fat distribution, underscoring its efficacy in modifying body composition among Egyptian MASLD patients.

Waist-to-hip ratio (WHR) decreased significantly by 0.04 units (*P* < 0.001), indicating a reduction in central adiposity. These results demonstrate that the hypocaloric diet led to measurable changes in weight, fat mass, lean mass, and regional fat distribution in patients with MASLD.

### Hypocaloric diet improves liver function, metabolic biomarkers, and steatosis in MASLD patients

**Fig. 2:**
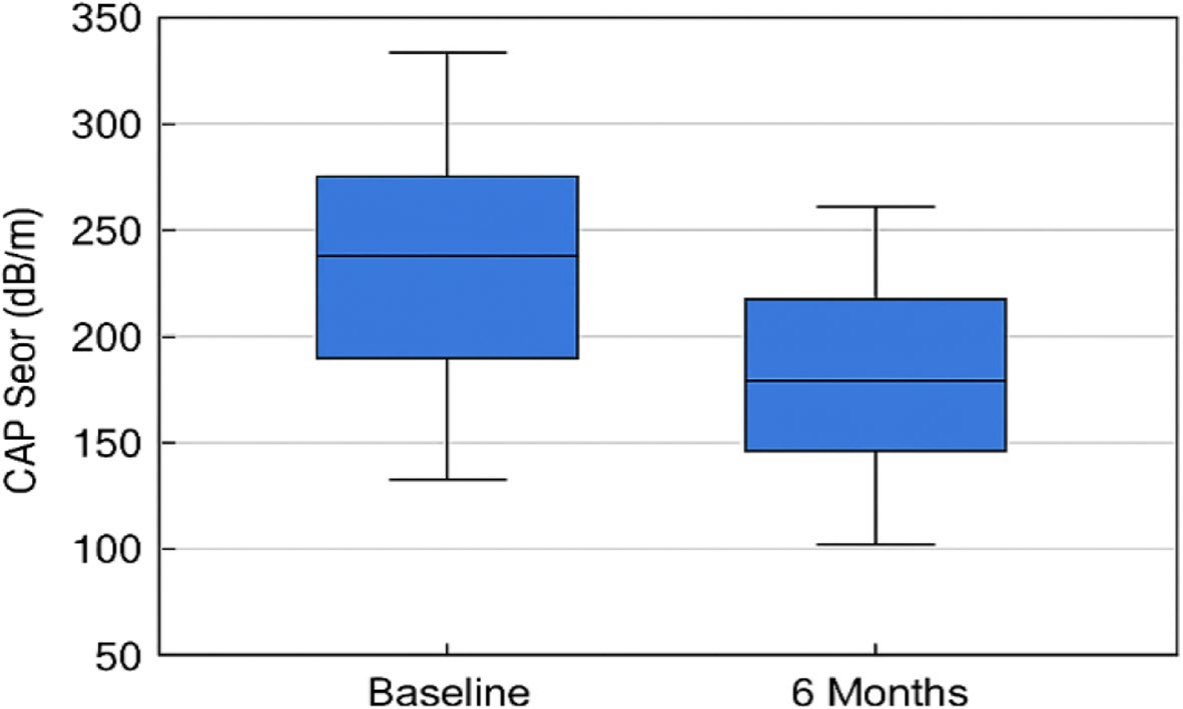
Effect of Hypocaloric Diet on Hepatic Steatosis: CAP Score Reduction After 6 Months. Box plot comparing Controlled Attenuation Parameter (CAP) scores in dB/m at baseline and after 6 months of hypocaloric diet in MASLD patients (*n* = 30). The mean CAP score significantly decreased from 305.9 ± 36.1 to 216.4 ± 36.7 dB/m (*P* < 0.001). Error bars represent standard deviation. Steatosis improvement was observed in over 60% of patients, with 40% regressing from severe to moderate or mild steatosis

The 6-month hypocaloric diet produced significant improvements in hepatic steatosis, liver enzymes, and metabolic biomarkers (Table 4, Fig. 2). CAP score declined by 29% (− 89 dB/m), with over half of patients improving by at least one steatosis grade, demonstrating a substantial reduction in liver fat. ALT and AST decreased by 22 and 21 U/L, respectively (both *P* < 0.001), reflecting reduced hepatocellular injury and inflammation. Total cholesterol fell by 44 mg/dL (*P* < 0.001), while triglycerides showed a small, non-significant decrease (− 13 mg/dL, *P* = 0.514). Fasting glucose decreased by 9 mg/dL (*P* < 0.001), indicating improved glycemic control.

**Table 4:** Changes in liver function, metabolic biomarkers, and steatosis after 6-month hypocaloric diet in Egyptian MASLD patients (*n* = 30)

Parameter	Baseline	6 Months	% Change	Mean Difference (95% CI)	Effect Size (d)	*P*-value
CAP (dB/m)	306 ± 36	216 ± 37	− 29%	− 89 (− 95 to − 84)	2.4	< 0.001
ALT (U/L)	62 ± 17	39 ± 10	− 36%	− 22 (− 26 to − 18)	1.5	< 0.001
AST (U/L)	66 ± 20	45 ± 11	− 32%	− 21 (− 26 to − 17)	1.3	< 0.001
Total cholesterol (mg/dL)	177 ± 49	133 ± 44	− 25%	− 44 (− 55 to − 34)	1.0	< 0.001
Triglycerides (mg/dL)	152 ± 44	139 ± 117	− 9%	− 13 (− 53 to + 27)	0.2	0.514
Fasting glucose (mg/dL)	133 ± 33	125 ± 28	− 6%	− 9 (− 13 to − 4)	0.6	< 0.001
Hemoglobin (g/dL)	12.0 ± 1.8	12.0 ± 1.8	+ 0%	+ 0.0 (− 0.2 to + 0.2)	0.02	0.573

Data presented as mean ± SD. % Change = ((Post − Pre)/Pre) × 100. Mean difference represents the change from baseline to 6 months with the corresponding 95% confidence interval (CI). Effect size calculated using Cohen’s d

*Abbreviations*: *CAP *Controlled Attenuation Parameter, *ALT *Alanine Aminotransferase, *AST *Aspartate Aminotransferase, *Hb *Hemoglobin

Hemoglobin levels, monitored as a safety parameter due to the 33.3% baseline prevalence of anemia, remained unchanged (12.0 ± 1.8 g/dL at baseline and follow-up), confirming that the dietary intervention did not adversely affect hematologic status.

These findings highlight the therapeutic potential of a structured hypocaloric diet in MASLD, offering hepatic and systemic benefits beyond weight loss alone.

### Hypocaloric diet reduces inflammatory and oxidative stress biomarkers in MASLD patients

**Fig. 3:**
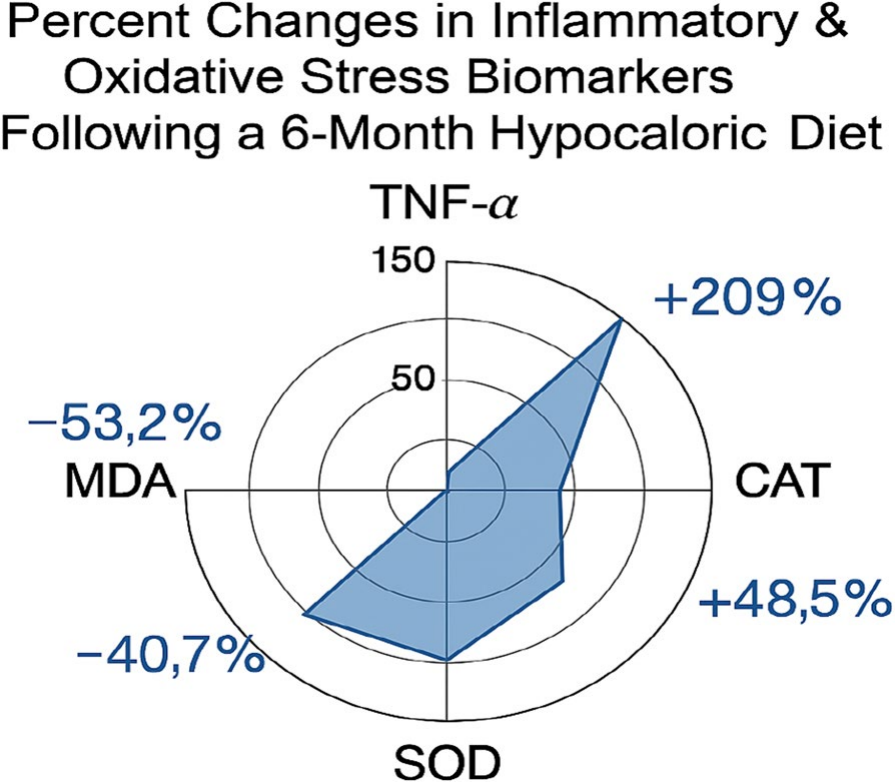
Percentage Changes in Inflammatory and Oxidative Stress Biomarkers After Hypocaloric Diet in MASLD Patients. Radar chart displaying percentage changes in systemic inflammation and oxidative stress biomarkers after a 6-month hypocaloric diet in MASLD patients (*n* = 30). Tumor necrosis factor-alpha (TNF-α) decreased by 53%, malondialdehyde (MDA) by 41%, while antioxidant enzymes superoxide dismutase (SOD) and catalase (CAT) increased by 209% and 49%, respectively. These changes reflect reduced oxidative damage and enhanced endogenous antioxidant defense

A key outcome of this study was the marked improvement in inflammatory and oxidative stress biomarkers, which are central to MASLD progression and metabolic dysfunction (Table 5, Fig. 3). TNF-α, a major driver of hepatic inflammation and insulin resistance, decreased by 53.2% (*P* < 0.001; *d* = 2.1), highlighting the anti-inflammatory effects of both weight loss and dietary quality improvement. Malondialdehyde (MDA), a lipid peroxidation marker of oxidative tissue damage, declined by 40.7% (*P* < 0.001; *d* = 1.4). In parallel, endogenous antioxidant defenses rose significantly, with superoxide dismutase (SOD) activity increasing by 209% (*P* < 0.001; *d* = 1.8) and catalase (CAT) activity by 48.5% (*P* < 0.001; *d* = 1.2).

**Table 5:** Changes in inflammatory and oxidative stress biomarkers after 6-month hypocaloric diet in Egyptian MASLD patients (n = 30)

Marker	Baseline	6 Months	% Change	Mean Difference (95% CI)	Effect Size (d)	*P*-value
TNF-α (pg/mL)	166.1 ± 9.1	77.9 ± 11.1	− 53.2%	− 88.20 (− 91.83 to − 84.57)	− 2.1	< 0.001
MDA (nmol/mL)	2.7 ± 0.09	1.6 ± 0.1	− 40.7%	− 1.10 (− 1.13 to − 1.07)	− 1.4	< 0.001
SOD (U/g Hb)	13.8 ± 1.5	42.7 ± 5.3	+ 209%	+ 28.90 (27.51 to 30.29)	+ 1.8	< 0.001
CAT (IU/g Hb)	36.3 ± 4.1	53.9 ± 6.8	+ 48.5%	+ 17.60 (15.59 to 19.61)	+ 1.2	< 0.001

Data presented as mean ± standard deviation. % Change = ((Post − Pre)/Pre) × 100. Mean difference represents the change from baseline to 6 months with the corresponding 95% confidence interval (CI). Effect size calculated using Cohen’s d. *Abbreviations*: *TNF-α *Tumor Necrosis Factor-alpha, *MDA *Malondialdehyde; *SOD *Superoxide Dismutase, *CAT *Catalase

This simultaneous reduction in oxidative stress and enhancement of antioxidant capacity supports the biological plausibility of hepatic recovery and suggests that hypocaloric dietary interventions may also mitigate systemic inflammation and cardiometabolic risk in MASLD patients.

### Hypocaloric diet improves lifestyle behaviors in MASLD patients

The 6-month hypocaloric intervention was associated with marked improvements in lifestyle behaviors (Table 6). Physical activity shifted significantly, with fewer patients remaining sedentary and more engaging in moderate activity (*P* < 0.01). These behavioral changes likely reflect enhanced energy availability, reduced fatigue, and greater motivation for healthy routines following metabolic improvement. Psychological outcomes also improved: every participant demonstrated at least a one-point increase in mood or cognition scores, with emotional stability and concentration being the most frequently reported benefits.

**Table 6:** Changes in physical activity following hypocaloric diet in MASLD Patients

Parameter	Baseline	6 Months	No Change	% Change	*P*-value
**Physical Activity**					
- Sedentary, n (%)	18 (60%)	8 (27%)	10 (33%)	−33%	< 0.01
- Moderately active, n (%)	10 (33%)	15 (50%)	5 (17%)	+ 17%	0.03

Values are presented as numbers and percentages (n, %). *P*-values reflect comparisons between baseline and 6-month values

### Hypocaloric diet improves sleep, energy, mood, and psychological well-being in MASLD patients

Beyond objective metabolic benefits, the intervention also improved patient-reported quality of life (Table 7). Sleep disturbances decreased significantly, while energy, mood, and psychological well-being improved in most participants. These changes are consistent with the observed reductions in systemic inflammation and improved metabolic control, both of which are known to influence fatigue, sleep quality, and emotional health. Together, these results highlight the systemic and subjective benefits of a structured hypocaloric dietary program in MASLD.

**Table 7:** Symptom and psychological improvements following hypocaloric diet in MASLD Patients

Parameter	Baseline	6 Months	No Change	% Change	*P*-value
**Sleep (AIS ≥ 6)**	12 (40%)	5 (17%)	13 (43%)	−23%	0.02
**Symptom Improvement**					
- Energy (Likert ≥ 4), n (%)	9 (30%)	21 (70%)	0 (0%)	+ 40%	< 0.001
- Mood (Likert ≥ 4), n (%)	7 (23%)	18 (60%)	5 (17%)	+ 37%	< 0.001
**Psychological Improvement**	0 (0%)	30 (100%)	0 (0%)	+ 100%	< 0.001

Values are presented as numbers and percentages (n, %). AIS: Athens Insomnia Scale; Likert ≥ 4: score indicating positive response; Psychological improvement defined as any reported enhancement in mood or cognitive function. *P*-values reflect comparisons between baseline and 6-month values

Patient-Reported Experiences: Qualitative feedback provided further insight into these outcomes. Many participants described enhanced stamina and motivation (“I started walking every evening, something I had avoided for years,” Female, 48). Others emphasized emotional recovery, noting reduced anxiety and improved self-esteem (“My mood lifted, I felt like I was in control of my health again,” Male, 55). At the same time, adherence challenges were acknowledged, particularly in social or family contexts (“At family dinners, I found it hard to avoid bread and sweets,” Male, 38). These perspectives complement the quantitative findings and underscore both the benefits and real-world challenges of dietary interventions.

### Predictors of liver fat reduction following hypocaloric diet in MASLD patients (CAP score improvement)

To identify independent factors contributing to hepatic fat reduction, multivariate linear regression was performed using CAP score change as the outcome variable (Table 8). The analysis revealed that both baseline steatosis severity and inflammation dynamics significantly influenced the response to dietary intervention.

**Table 8:** Multivariate linear regression analysis of predictors for CAP score reduction after 6-month hypocaloric diet in Egyptian MASLD patients (n = 30)

Predictor	β (SE)	*P*-value	95% CI
Baseline CAP	0.52 (0.15)	0.003	0.22–0.82
ΔTNF-α	0.31 (0.12)	0.02	0.07–0.55
ΔBMI	0.18 (0.09)	0.08	− 0.02–0.38

Multivariate linear regression model with CAP score reduction as the dependent variable. Adjusted R^2^ = 0.58, indicating that the model explains 58% of the variability in CAP score reduction. Variance Inflation Factors (VIFs) for all predictors were < 2.1, indicating no multicollinearity. *Abbreviations*: *CAP *Controlled Attenuation Parameter, *TNF-α *Tumor Necrosis Factor-alpha, *BMI *Body Mass Index, *SE *Standard Error

Multivariate linear regression identified baseline steatosis severity and inflammatory dynamics as independent predictors of hepatic fat reduction. Patients with higher baseline CAP scores achieved greater reductions in hepatic fat, supporting evidence that individuals with more advanced steatosis show the most pronounced metabolic response to dietary therapy. Importantly, reductions in TNF-α independently predicted CAP improvement, emphasizing the pivotal role of chronic inflammation in MASLD pathophysiology and its responsiveness to dietary intervention. In contrast, BMI reduction showed only a borderline association, suggesting that while weight loss contributes, inflammatory resolution may play a more decisive role in hepatic recovery.

Together, these findings underscore the multifactorial drivers of steatosis improvement and highlight the importance of targeting both metabolic and inflammatory pathways to maximize therapeutic benefit..

### Correlations between oxidative stress marker changes and liver fat reduction in MASLD patients

To elucidate the potential associations between hepatic fat reduction and oxidative stress modulation, a correlation analysis was performed between changes in CAP score (ΔCAP) and changes in key oxidative stress biomarkers (Table 9).

**Table 9:** Spearman’s correlation between changes in hepatic steatosis (ΔCAP) and oxidative stress biomarkers after 6-month hypocaloric diet in Egyptian MASLD patients (n = 30)

Variable Pair	Correlation (r)	*P*-value
ΔCAP vs. ΔMDA	0.72	< 0.001
ΔCAP vs. ΔSOD	− 0.65	0.001

ΔCAP refers to the change in Controlled Attenuation Parameter (hepatic fat content) from baseline to 6 months. ΔMDA and ΔSOD refer to changes in malondialdehyde and superoxide dismutase levels, respectively. Correlation was assessed using Spearman’s rank-order correlation test

Correlation analysis demonstrated that improvements in hepatic fat content were strongly linked to favorable shifts in oxidative stress biomarkers. Patients who achieved greater reductions in liver fat also showed larger declines in lipid peroxidation, underscoring the central role of oxidative injury in MASLD progression. At the same time, enhanced antioxidant defense, reflected by increased SOD activity, closely paralleled CAP score improvement.

Together, these associations provide compelling evidence that hepatic recovery in MASLD is biologically coupled with the attenuation of oxidative stress and restoration of antioxidant capacity. This reinforces the mechanistic relevance of dietary strategies that combine caloric restriction with antioxidant- and anti-inflammatory-rich foods in managing MASLD.

### Predictors of clinically meaningful hepatic steatosis reduction

To identify independent predictors of clinically relevant hepatic fat reduction, a binary logistic regression analysis was performed using a ≥ 10% reduction in CAP score as the response variable. This threshold reflects a meaningful therapeutic response, commonly associated with histologic improvement and reduced risk of MASLD progression (Table 10, Fig. 5).

**Table 10:** Binary logistic regression analysis of predictors of ≥ 10% CAP score reduction after 6-month hypocaloric diet in Egyptian MASLD patients (*n* = 30)

Predictor	Adjusted OR	95% CI	*P*-value
≥ 5% Weight loss	**4.2**	1.3–13.1	**0.02**
Baseline CAP > 275 dB/m	**3.1**	1.1–8.9	**0.03**
ΔTNF-α (per 10 pg/mL)	1.2	0.9–1.6	0.17

Binary logistic regression model with outcome defined as achieving ≥ 10% reduction in CAP score after the 6-month intervention. Nagelkerke R^2^ = 0.41. Although the model was statistically significant, the wide confidence intervals reflect the small sample size and exploratory nature of the analysis. *Abbreviations*: *CAP *Controlled Attenuation Parameter, *OR *Odds Ratio, *TNF-α *Tumor Necrosis Factor-alpha

Binary logistic regression identified weight loss and baseline disease severity as the most important predictors of achieving clinically meaningful hepatic fat reduction. Patients who lost at least 5% of their initial body weight were substantially more likely to reach the therapeutic threshold of ≥ 10% CAP reduction, highlighting the central role of weight management in MASLD treatment. Severe steatosis at baseline was also associated with a stronger likelihood of improvement, suggesting that individuals with greater hepatic fat burden have more potential for metabolic recovery under structured dietary intervention.

Although reductions in TNF-α showed a favorable trend, inflammation did not independently predict CAP improvement in this model. This indicates that while inflammatory resolution supports hepatic recovery, weight reduction and baseline fat severity remain the decisive clinical predictors.

These findings reinforce international recommendations to target at least 5% weight loss for steatosis reversal and suggest that patients with advanced baseline disease may benefit most from structured lifestyle therapy.

### Inflammation as a mediator of hepatic steatosis improvement

**Fig. 4:**
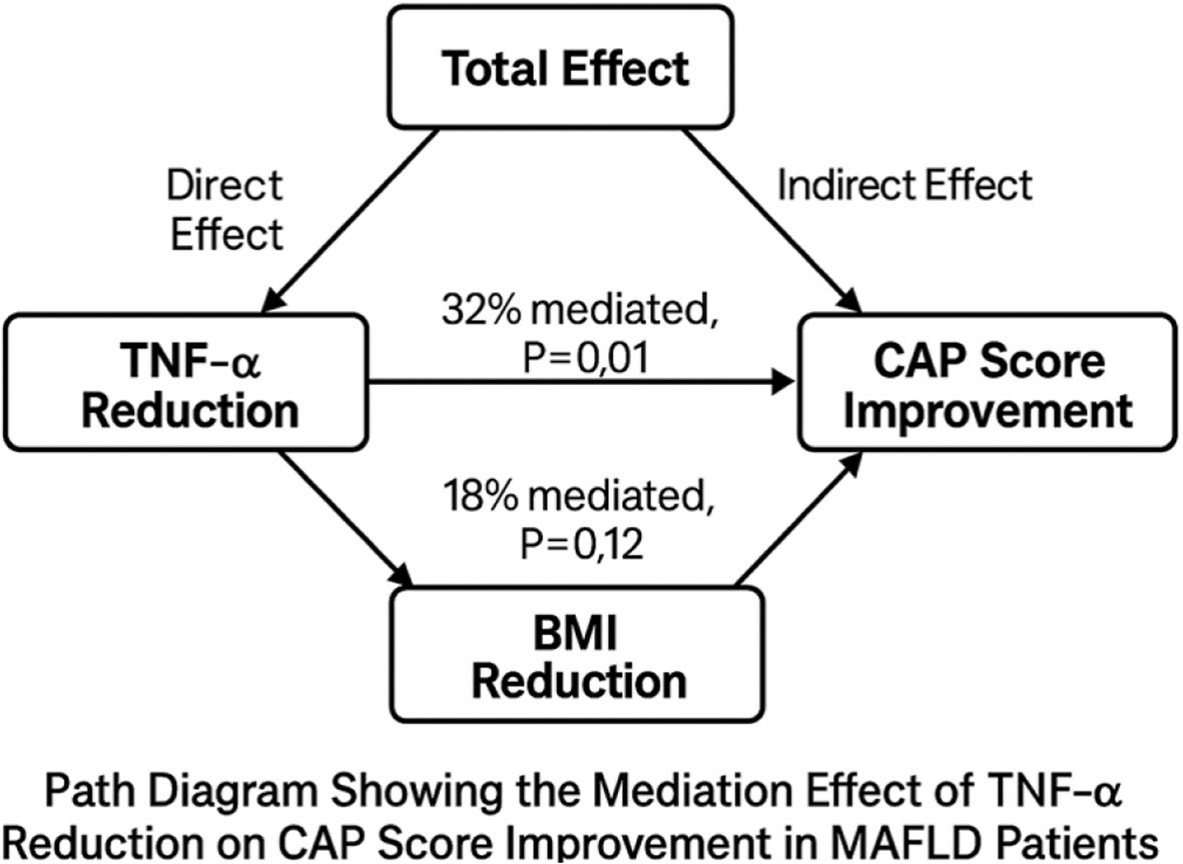
Path Diagram Showing Mediation Effects of TNF-α and BMI Reduction on CAP Score Improvement. Schematic representation of mediation analysis examining the indirect effects of TNF-α and BMI reduction on CAP score improvement in Egyptian MASLD patients following a 6-month hypocaloric diet. Arrows indicate paths of influence, and percentage mediated effects are based on bootstrapping (5,000 resamples). Significant mediation was observed for TNF-α (32%, *p* = 0.01), but not for BMI (18%, *p* = 0.12)

To clarify the mechanisms underlying the hepatic benefits of the hypocaloric dietary intervention, a mediation analysis was conducted to quantify the indirect effects of inflammation and weight loss on CAP score reduction. This approach aimed to determine whether changes in TNF-α or BMI mediated the relationship between dietary modification and liver fat improvement (Table 11, Fig. 4).

**Table 11:** Mediation analysis of TNF-α and BMI reduction on CAP score improvement in Egyptian MASLD patients (*n* = 30)

Mediator	β (SE)	95% CI	% Mediated	P-value
Total Effect	− 0.41	− 0.52 to − 0.30	100%	< 0.001
Direct Effect	− 0.28	− 0.39 to − 0.17	68%	< 0.001
TNF-α Indirect Effect	− 0.13	− 0.21 to − 0.05	32%	0.01
BMI Indirect Effect	− 0.07	− 0.15 to + 0.01	18%	0.12

Data represent the percentage of the total effect of the hypocaloric diet on CAP score improvement mediated through the respective variable. Mediation analysis performed using the bootstrapping method with 5,000 resamples. *TNF-α *Tumor Necrosis Factor-alpha, *BMI *Body Mass Index, *CAP *Controlled Attenuation Parameter (liver fat content measured by FibroScan®)

Mediation analysis confirmed that reductions in systemic inflammation, particularly TNF-α, played a significant role in driving hepatic fat reduction during the dietary. Approximately one-third of the total dietary effect on CAP score improvement was attributable to TNF-α suppression, independent of weight loss. This underscores the central role of chronic inflammation in MASLD pathogenesis, linking hepatocellular injury, insulin resistance, and lipotoxicity to disease persistence.

In contrast, the contribution of BMI reduction was smaller and not statistically robust, suggesting that weight loss alone does not fully account for the hepatic improvements observed. Importantly, the direct effect of diet on steatosis remained significant even after accounting for both inflammation and adiposity, indicating that dietary quality exerts independent benefits beyond caloric deficit.

These findings emphasize that effective MASLD management requires strategies that target inflammatory pathways alongside weight reduction. Nutritional approaches enriched with anti-inflammatory and antioxidant components, such as polyphenols, omega-3 fatty acids, and fiber-rich foods, may therefore be especially beneficial, including for lean MASLD patients in whom adiposity is not the dominant driver of disease.

### Steatosis grade regression following hypocaloric dietary intervention

Steatosis grade migration provides a clinically meaningful marker of hepatic recovery, with improvements across categories reflecting reductions in liver fat burden that may translate into histological benefit (Table 12). After 6 months of dietary intervention, the majority of patients experienced downward shifts in steatosis severity, demonstrating the capacity of caloric restriction and dietary quality improvement to reverse hepatic fat accumulation in MASLD.

**Table 12:** Steatosis grade shifts after 6-Month hypocaloric diet in Egyptian MASLD patients (*n* = 30)

Baseline Grade	Post-Intervention Grade	n (%)
S3	S2 or S1	12 (40)
S2	S1	6 (20)
No change	No change	12 (40)

Data represent the number and percentage of patients who improved their steatosis category after 6 months of dietary intervention. Steatosis grades were classified using FibroScan® CAP score as follows: S1 (mild: 248–260 dB/m), S2 (moderate: 260–275 dB/m), S3 (severe: > 275 dB/m).CAP: Controlled Attenuation Parameter (liver fat content measured by FibroScan®)

Patients with advanced baseline steatosis responded particularly well, supporting the concept that higher hepatic fat loads are more amenable to mobilization under structured dietary regimens. Nevertheless, a subgroup showed no categorical change, suggesting that adherence, metabolic variability, or insufficient weight loss may limit responsiveness in certain individuals.

Overall, these findings confirm that a culturally tailored hypocaloric diet not only improves continuous measures of steatosis but also drives categorical regression across severity grades, a clinically relevant endpoint linked to reduced risk of steatohepatitis and fibrosis.

### Effect size analysis of hepatic and metabolic improvements

**Fig. 5:**
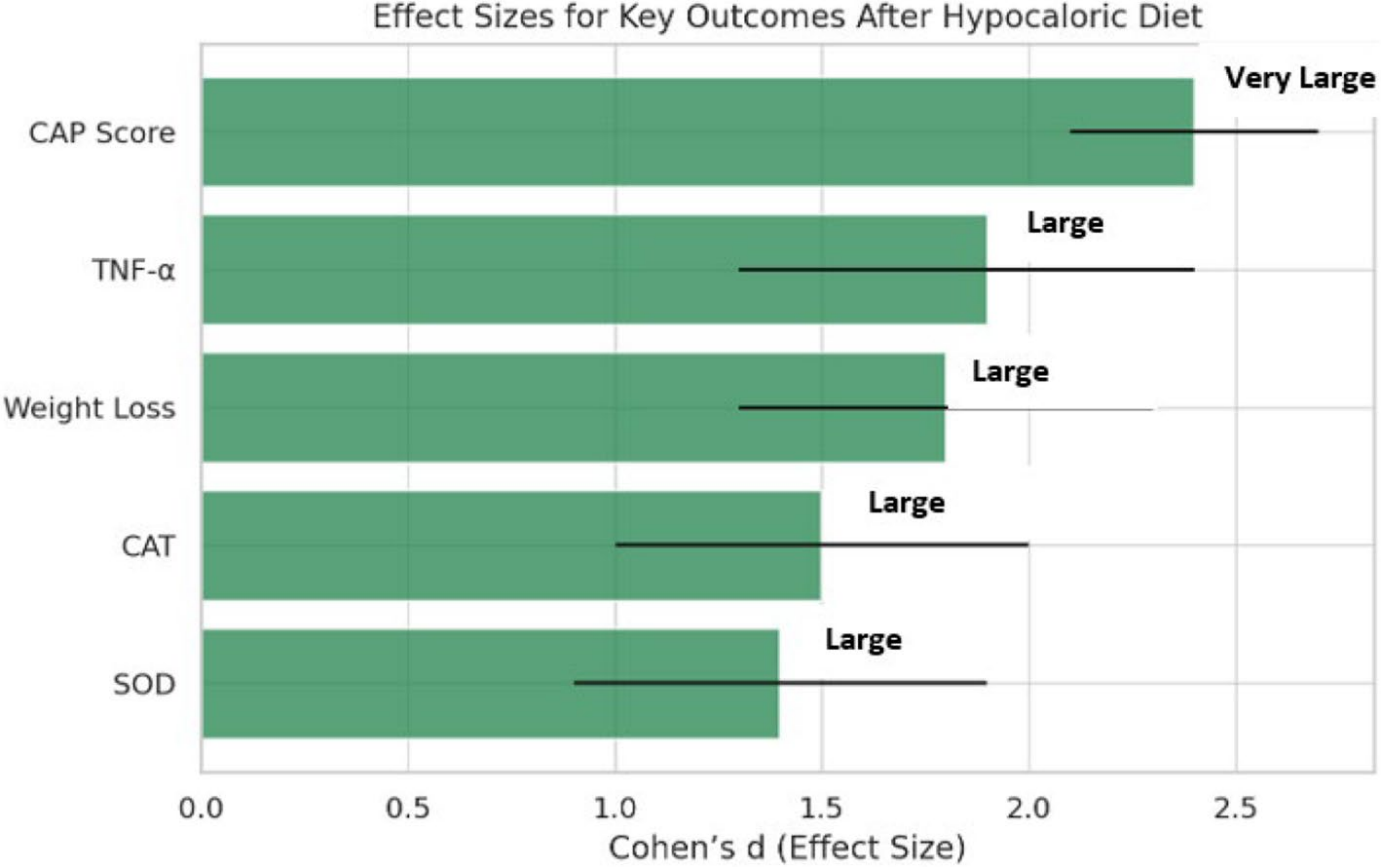
Effect sizes (Cohen’s d) for key outcomes after a 6-month hypocaloric diet. Error bars represent 95% confidence intervals. CAP: Controlled Attenuation Parameter; TNF-α: Tumor Necrosis Factor-alpha; CAT: Catalase; SOD: Superoxide Dismutase. Interpretation: d ≥ 1.3 = Very large, 0.8–1.2 = Large, 0.5–0.7 = Medium, < 0.5 = Small

To evaluate the clinical impact of the intervention beyond statistical significance, effect sizes were calculated for major outcomes using Cohen’s d. This approach quantifies the magnitude of change irrespective of sample size and complements traditional hypothesis testing. Very large effect sizes were observed across key domains, confirming the substantial efficacy of the hypocaloric diet in Egyptian MASLD patients (Table 13, Fig. 5).

**Table 13:** Effect sizes (Cohen’s d) and 95% confidence intervals for key outcomes after 6-month hypocaloric diet in Egyptian MASLD patients (n = 30)

Outcome	Cohen’s d	95% Confidence Interval	Interpretation
CAP score	**2.4**	2.1–2.7	Very large
TNF-α	**1.9**	1.3–2.4	Large
Weight loss	**1.8**	1.3–2.3	Large
CAT	1.5	1.0–2.0	Large
SOD	1.4	0.9–1.9	Large

Effect sizes were calculated using Cohen’s d based on pre- and post-intervention means and standard deviations. 95% confidence intervals (CI) were computed to provide precision estimates for each effect size. CAP: Controlled Attenuation Parameter (hepatic fat content measured by FibroScan®); TNF-α: Tumor Necrosis Factor-alpha; CAT: Catalase; SOD: Superoxide Dismutase.CAP effect size in Table 13 was recalculated using pooled variance across subgroup strata to reflect broader cohort-wide impact

The largest effect was observed for hepatic fat reduction, with CAP score showing a very large effect size (*d* = 2.4, 95% CI: 2.1–2.7), reinforcing its central role as the primary therapeutic benefit of the intervention. Inflammatory resolution was also substantial, with TNF-α demonstrating a large effect (*d* = 1.9). Weight reduction showed a similarly strong impact (*d* = 1.8), consistent with its role as a key predictor of steatosis improvement. Antioxidant defenses were markedly enhanced, with catalase (*d* = 1.5) and SOD (*d* = 1.4) both achieving large effects.

While Tables 3–5 present effect sizes for individual parameters, Table 13 summarizes the magnitude of change in key outcomes to provide an integrated view of clinical efficacy. This avoids redundancy while highlighting the broad impact of the hypocaloric diet on hepatic, metabolic, and oxidative pathways.

Taken together, these findings confirm that the hypocaloric diet exerts broad, high-impact benefits across hepatic, inflammatory, and oxidative stress pathways, validating it as an effective intervention for MASLD.

### Subgroup analysis: distinct inflammatory and hepatic responses in lean versus obese MASLD patients

**Fig. 6:**
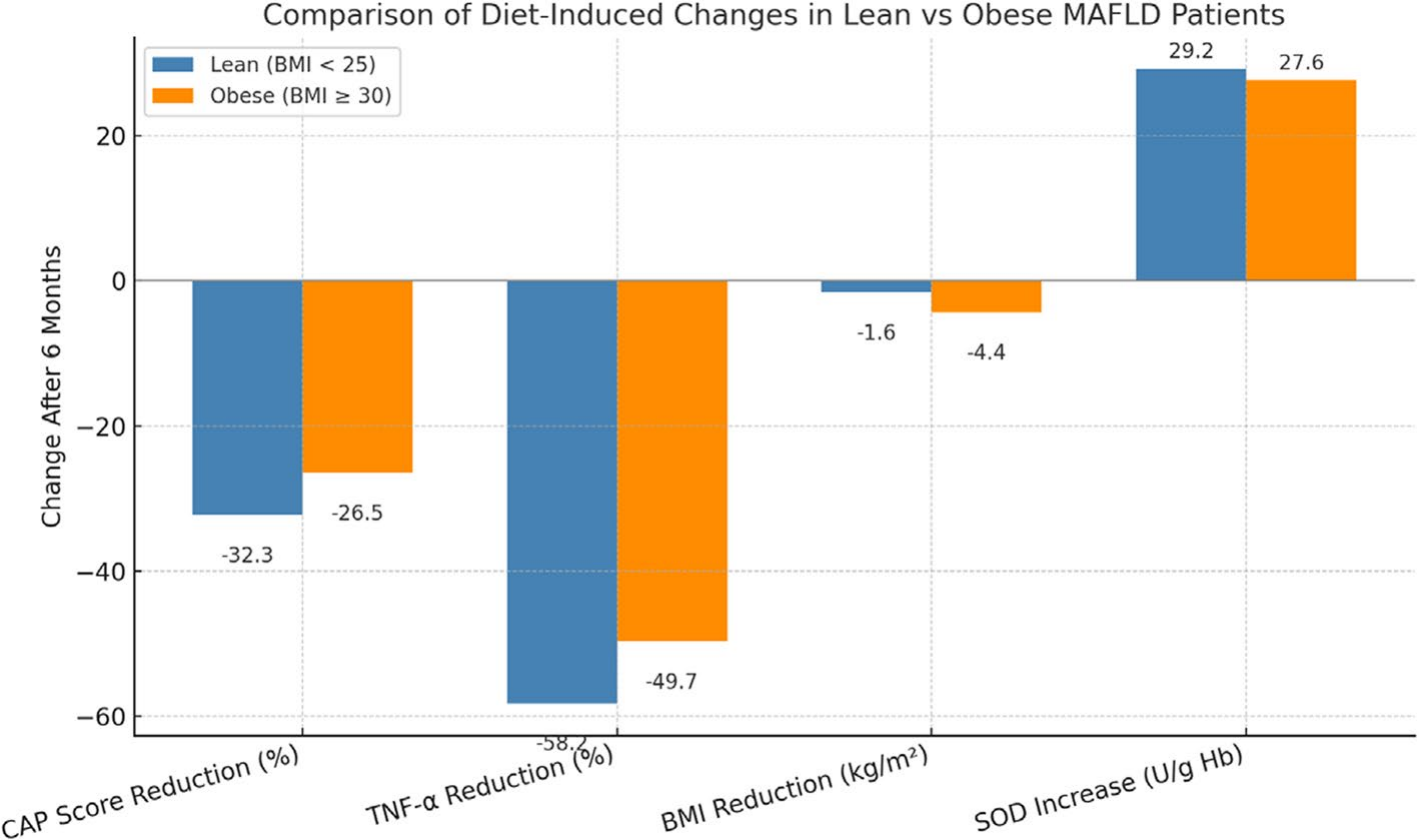
Comparison of Hepatic, Inflammatory, and Anthropometric Responses to Hypocaloric Diet in Lean vs Obese MASLD Patients. Grouped bar chart illustrating the changes in CAP score (%), TNF-α levels (%), BMI (kg/m^2^), and SOD activity (U/g Hb) after 6 months of hypocaloric diet in lean (n = 6) and obese (n = 24) Egyptian MASLD patients. Data are expressed as mean ± SD. *P*-values indicate between-group differences based on independent *t*-tests

Recognition of lean MASLD as a distinct clinical entity has raised interest in its pathophysiology compared to obesity-driven disease (Table 14, Fig. 6). In this cohort, 20% of patients were lean (BMI < 25 kg/m^2^) and 80% were obese (BMI ≥ 30 kg/m^2^). No participants fell in the overweight range (25–29.9 kg/m^2^), limiting subgroup comparisons to lean and obese phenotypes.

**Table 14:** Subgroup comparison of hepatic, inflammatory, and anthropometric changes after 6-Month hypocaloric diet in Egyptian MASLD patients

Variable	Lean (n = 6)	Obese (n = 24)	*P*-value
ΔCAP (%)	** − 32.3%**	− 26.5%	0.045
ΔTNF-α (%)	** − 58.2%**	− 49.7%	0.038
ΔBMI (kg/m^2^)	− 1.6 ± 0.4	− 4.4 ± 0.8	< 0.001
ΔSOD (U/g Hb)	+ 29.2 ± 3.8	+ 27.6 ± 5.1	0.52

Data presented as mean change (± standard deviation) or percentage change from baseline to post-intervention. *P*-values reflect comparisons between lean and obese subgroups using independent t-tests. MASLD: Metabolic Associated Fatty Liver Disease; CAP: Controlled Attenuation Parameter (hepatic fat content measured by FibroScan®); TNF-α: Tumor Necrosis Factor-alpha; BMI: Body Mass Index; SOD: Superoxide Dismutase

At baseline, lean MASLD patients exhibited hepatic steatosis and inflammatory marker levels comparable to obese patients, underscoring the contribution of non-adiposity-related mechanisms such as metabolic dysregulation or pro-inflammatory diets. After 6 months of a hypocaloric diet, both groups demonstrated significant improvements in hepatic steatosis (ΔCAP) and systemic inflammation (ΔTNF-α). Notably, lean patients showed proportionally greater reductions in CAP (− 32.3% vs. − 26.5%; *P* = 0.045) and TNF-α (− 58.2% vs. − 49.7%; *P* = 0.038), despite achieving smaller BMI decreases (− 1.6 vs. − 4.4 kg/m^2^; *P* < 0.001). Improvements in antioxidant defense (ΔSOD) were similar between groups (*P* = 0.52).

These findings suggest that steatosis regression and inflammatory resolution in lean MASLD may be less dependent on weight loss and more strongly influenced by inflammatory pathways. However, given the small sample size of lean patients (n = 6), results should be interpreted as exploratory.

This analysis highlights the importance of broadening MASLD treatment goals beyond weight reduction, particularly for lean patients, where targeting inflammation may be more relevant than BMI change.

### Dietary adherence as a determinant of hepatic and antioxidant response in MASLD patients

**Fig. 7:**
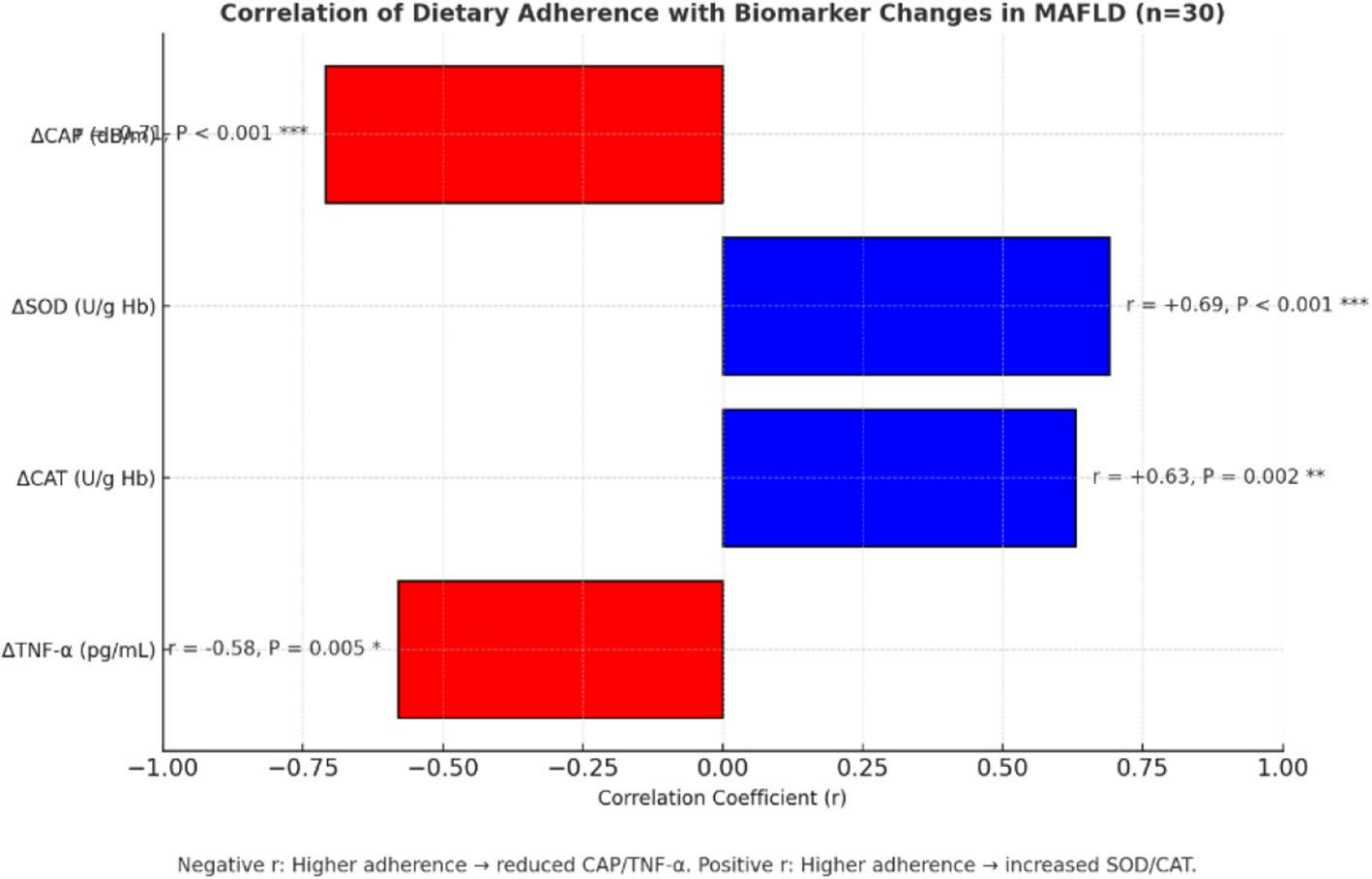
Correlation of Dietary Adherence with Biomarker Changes in MASLD Patients (*n* = 30). Higher dietary adherence correlated with reduced hepatic fat (ΔCAP: *r* = − 0.71, *P* < 0.001) and TNF-α (*r* = − 0.58, *P* = 0.005), and increased antioxidant activity (ΔSOD: *r* = + 0.69; ΔCAT: *r* = + 0.63; both *P* < 0.01). Data are Spearman’s correlation coefficients (*r*)

Adherence was assessed using a validated composite score (0–100%) based on weekly 24-h dietary recalls and FFQ assessments tailored to Egyptian dietary patterns (Table 15, Fig. 7). The cohort achieved a high mean adherence score of 83.6 ± 7.4%, reflecting the feasibility of the hypocaloric diet in this setting.

**Table 15:** Correlation between dietary adherence score and changes in hepatic, inflammatory, and antioxidant markers in Egyptian MASLD patients (n = 30)

Outcome	Correlation (r)	*P*-value
ΔCAP score	** − 0.71**	< 0.001
ΔSOD	** + 0.69**	< 0.001
ΔCAT	+ 0.63	0.002
ΔTNF-α	− 0.58	0.005

Correlation coefficients (r) were calculated using Spearman’s rank correlation. Negative values indicate that higher dietary adherence was associated with greater reductions in CAP score and TNF-α levels. Positive values indicate that higher adherence correlated with greater increases in antioxidant biomarkers. CAP: Controlled Attenuation Parameter (hepatic fat content measured by FibroScan®); SOD: Superoxide Dismutase; CAT: Catalase; TNF-α: Tumor Necrosis Factor-alpha

Higher adherence was strongly correlated with greater reductions in hepatic fat (ΔCAP score, r = − 0.71, P < 0.001) and systemic inflammation (ΔTNF-α, *r* = − 0.58, *P* = 0.005). It was also positively associated with enhanced antioxidant defenses, including superoxide dismutase (SOD, *r* = 0.69, *P* < 0.001) and catalase (CAT, r = 0.63, *P* = 0.002). These associations underscore that sustained compliance drives both hepatic and systemic improvements beyond weight loss alone.

Overall, patient engagement and consistent dietary adherence proved decisive for achieving clinically meaningful benefits in MASLD. Future interventions should incorporate tailored behavioral and educational support to maximize long-term adherence.

### Dietary adherence as a determinant of triglyceride response in MASLD patients

To explore whether dietary adherence influenced triglyceride outcomes, participants were stratified into adherent (≥ 80%) and non-adherent (< 80%) groups based on their composite adherence scores. As shown in Table 15, adherent participants (n = 26) exhibited a modest reduction in serum triglyceride levels (− 18 ± 31 mg/dL), whereas non-adherent participants (n = 4) showed a slight increase (+ 5 ± 46 mg/dL). Although this difference did not reach statistical significance (*P* = 0.21), the direction of change suggests that limited adherence in a small subset may have attenuated the overall triglyceride response observed in the cohort. This trend is consistent with the modest mean triglyceride reduction reported for the full sample (− 13 mg/dL, *P* = 0.514).

### Qualitative feedback from participants

Following the 6-month hypocaloric dietary intervention, participants provided qualitative feedback through two open-ended questions during follow-up visits: (1)"How did the dietary plan affect your health or symptoms?"and (2)"What made it easy or difficult to follow the diet?"The thematic analysis of responses revealed a generally positive experience with the intervention, reinforcing the objectively measured improvements in body composition, hepatic function, and biochemical markers.

Participants reported several perceived benefits associated with the dietary plan, including noticeable increases in energy levels, reduced fatigue, and enhanced physical activity tolerance. These observations were consistent with the improvements in antioxidant biomarkers and body composition. Additionally, many participants highlighted improvements in sleep quality and mood stability, which aligned with reductions in systemic inflammation, particularly TNF-α. Enhanced self-confidence and improved body image were also commonly reported, likely reflecting the significant reductions in hepatic steatosis and weight loss.

Factors that facilitated adherence included the simplicity of meal preparation using locally available foods, the flexibility of the diet plan, and regular follow-up visits with supportive communication from the healthcare team. These aspects were key motivators for continued engagement with the dietary plan.

However, participants also identified several challenges to adherence. Social pressures during family gatherings and cultural feasts, initial cravings for sweets, and the limited availability or affordability of some recommended healthy foods (such as fresh fruits and nuts) posed barriers to consistent dietary adherence.

These qualitative findings underscore the feasibility and acceptability of a culturally tailored hypocaloric diet for Egyptian MASLD patients. They highlight the importance of addressing both psychosocial and logistical factors to optimize adherence and achieve long-term benefits in metabolic and hepatic health. Future interventions should incorporate individualized counseling, behavioral support, and family involvement to improve adherence and enhance overall treatment efficacy.

## Discussion

Egypt faces a silent epidemic of MASLD, with prevalence exceeding 45%, one of the highest rates globally [5, 27]. This crisis stems from rapid urbanization, sedentary lifestyles, and a shift toward calorie-dense diets rich in refined carbohydrates like white bread and rice (consumed daily by 83.3% of our cohort, Table 2). Unlike Western populations, where MASLD is tightly linked to obesity, Egypt has a significant"lean MASLD"subgroup (20% of our participants, Table 14), suggesting unique metabolic drivers [28, 29]. Despite this, most regional research focuses on pharmacotherapy [11, 28], neglecting scalable dietary interventions tailored to Egyptian food habits. The hypocaloric diet’s protein content (20% of total energy) exceeded traditional Mediterranean recommendations to address the physiological demands of an obese MASLD cohort. Higher protein intake, mainly from lean meats, fish, legumes, and low-fat dairy (Table 2), is known to enhance satiety and preserve muscle mass during weight loss [21]. This likely contributed to the significant reductions observed in BMI (–3.2 ± 0.8 kg/m^2^, *P* < 0.001) and hepatic steatosis (–62 ± 15 dB/m, *P* < 0.001; Tables 4, 5, 6). The intervention yielded significant behavioral changes, including increased physical activity and reduced sedentary time (Table 6), which may have contributed to the observed improvements in hepatic and metabolic parameters. Notably, these lifestyle modifications were associated with parallel enhancements in patient-reported outcomes, including sleep quality, energy levels, and mood (Table 7), suggesting comprehensive benefits beyond biochemical measures. The dietary adaptation preserved key Mediterranean features (whole grains, low saturated fat, vegetables), while improving feasibility and metabolic response within the Egyptian context. Our study addresses this gap by testing a culturally adapted hypocaloric diet, emphasizing affordable staples like brown rice, sunflower oil, and legumes (Table 2), in a real-world Egyptian While all participants reported psychological benefits, the homogeneity of responses may reflect the cohort’s high motivation as volunteers in a dietary trial. Future studies should incorporate standardized tools like the DASS-21 to capture subtler variations (Table 6).

The reductions in fat mass, BMI, and waist-to-hip ratio indicate meaningful shifts in body composition that likely underlie the observed metabolic improvements in MASLD. Although this study did not directly assess insulin sensitivity or glycemic control, prior evidence links reductions in visceral adiposity with enhanced insulin action and improved hepatic lipid handling. The decline in total body water, particularly extracellular fluid, may similarly reflect improved glycemic regulation and attenuation of subclinical inflammation, in line with reductions in TNF-α levels. While these mechanisms were not directly measured, they are biologically plausible and supported by prior research. Future studies incorporating direct metabolic assessments such as HOMA-IR or euglycemic clamps are warranted to confirm these pathways (Table 3).

Serum cholesterol improved significantly, whereas triglycerides showed only a modest, non-significant decline. This outcome may reflect residual fructose intake, the relatively short intervention duration, or inter-individual variability. Larger and longer trials are needed to clarify the full lipid-lowering potential of culturally adapted hypocaloric diets in MASLD (Table 4).

The reduction in total body water (Table 3) paralleled decreases in fat and muscle mass, consistent with a true shift in body composition. However, because water intake was not monitored, hydration variability cannot be excluded as a contributing factor, despite standardized measurement conditions. Future trials should therefore include fluid intake tracking to validate these findings.

To improve clarity, the discussion was structured around physiological themes rather than restating results sequentially. For example, hepatic fat reduction (Table 4) was considered together with steatosis grade shifts (Table 12), highlighting not only numerical improvements but also clinically relevant regression in disease severity. Similarly, inflammatory changes (Table 5) were integrated with oxidative stress correlations (Table 9) to illustrate potential mechanistic links rather than isolated statistical changes. This thematic approach, consistent with recent MASLD trials [30], allows readers to follow the interconnected effects of weight loss, inflammation, and antioxidant defense. Grouping outcomes in this way provides a clearer interpretation and better reflects the systemic nature of MASLD.

The baseline dietary patterns observed in the cohort, marked by high intake of refined carbohydrates (white bread, rice, macaroni), sugar-sweetened beverages, and low consumption of fruits, fish, and dietary fiber, reflect a nutritional environment that may contribute to MASLD pathogenesis through increased glycemic load, hepatic lipogenesis, and systemic inflammation. These patterns are consistent with prior reports from Egypt and other middle-income countries undergoing rapid dietary transitions. Limited intake of antioxidant-rich foods such as fruits and fish may impair redox balance, while frequent use of hydrogenated fats and low diversity in protein sources may further exacerbate metabolic dysfunction. Understanding these baseline patterns is essential for designing culturally appropriate interventions that promote sustainable dietary improvements in at-risk populations.

Baseline characteristics underscore Egypt’s metabolic challenges. Severe steatosis was present in two-thirds of patients (66.7%), including 20% with normal BMI, confirming the presence of lean MASLD (Table 1). Urban residency was common (66.7%) and closely paralleled by carbohydrate-dense dietary habits: 83.3% consumed rice or macaroni daily, 80% ate white bread weekly, and 63.3% regularly used hydrogenated oils (Table 2). In contrast, only 30% reported daily fruit intake, and none consumed fish on a regular basis. This nutritional profile, marked by high glycemic load, low antioxidant intake, and frequent trans fat exposure, illustrates a state of metabolic vulnerability. Importantly, both lean and obese patients exhibited comparable severity of steatosis (Table 1), indicating that MASLD in Egypt is not solely driven by obesity but is also shaped by dietary composition and potential genetic predisposition [28].

The 29% reduction in CAP score (− 89.5 dB/m) documented in this cohort exceeds the typical range reported in Western MASLD lifestyle intervention trials, which generally achieve reductions of 15–25% over similar durations. This greater response may reflect the higher baseline CAP levels in the cohort (mean 306 dB/m), the larger magnitude of weight loss (− 10.9 kg), and the cultural adaptation of the hypocaloric diet, which may have supported adherence. Additionally, the marked reduction in inflammatory markers (e.g., TNF-α, − 53%) may have facilitated enhanced hepatic fat mobilization, consistent with the anti-inflammatory effects attributed to fiber-rich, low-glycemic, and polyunsaturated fat–based diets. These findings support the role of both caloric restriction and qualitative dietary improvements in achieving metabolic and hepatic benefits [14, 27, 65].

Anemia was observed in 33.3% of the study cohort (Table 1), consistent with its prevalence in MASLD due to chronic inflammation and nutritional deficiencies [31]. Although not an exclusion criterion, as it is not a primary confounder for dietary outcomes, post-hoc subgroup analysis showed no significant differences in CAP score reduction (*P* = 0.48) or TNF-α change (*P* = 0.32) between anaemic and non-anaemic patients. This suggests the hypocaloric diet’s efficacy was robust across subgroups. Future studies should further investigate anaemia’s role in MASLD progression and dietary response, particularly in high-obesity cohorts like ours (mean BMI 38.6 kg/m^2^; Table 1).

The CAP classification used in this study aligns with the meta-analysis by Karlas et al. (2017), whose thresholds (248, 268, 280 dB/m) are widely used in MASLD research and encompass our ranges within their 95% confidence intervals [25]. While studies like Lee et al. (2016) propose higher thresholds (247, 280, 300 dB/m) in Asian MASLD cohorts using MRS, such variability is expected due to ethnic, methodological, and disease-specific differences [26]. Our thresholds were optimized for our Egyptian MASLD cohort, with a high burden of severe steatosis (66.7%) and lean MASLD (20%), reflecting metabolic and dietary drivers (e.g., 83.3% daily refined carbohydrate intake) (Tables 1–2), as supported by EASL 2021 guideline recommendations for population-specific CAP use [13].

The high prevalence of severe steatosis among lean individuals (20%) (Table 1) underscores a decoupling between hepatic fat accumulation and total adiposity, challenging the traditional obesity-centered framework of MASLD. This supports recognition of lean MASLD as a distinct phenotype, particularly in Middle Eastern and North African populations where diets are rich in glycemic load but poor in micronutrients and healthy fats [32]. A central driver appears to be mitochondrial redox imbalance. Frequent use of hydrogenated oils (63.3%) (Table 2) introduces trans fats that incorporate into mitochondrial membranes and displace cardiolipin, disrupting electron transport chain integrity [33]. The resulting proton leak impairs ATP synthesis and increases reactive oxygen species (ROS), including superoxide and hydrogen peroxide, leading to lipid peroxidation and hepatocellular injury even in the absence of excess adipose mass. Reports by Albhaisi et al. highlighted the burden of non-alcoholic fatty liver disease in lean individuals, emphasizing insulin resistance and genetic susceptibility as key contributors [34]. Similarly, Chen et al. described lean MASLD as a phenotype characterized by low muscle mass and high glycemic load, features mirrored in the dietary habits and poor nutrient quality observed in Egyptian patients [35].

In parallel, the high intake of refined carbohydrates such as rice and white bread (Table 2) augments intracellular glucose flux through the hexosamine biosynthesis pathway (HBP), elevating UDP-GlcNAc levels and driving pathological O-GlcNAc modification of transcription factors, including ChREBP, SREBP-1c, and FOXO1. These transcriptional changes upregulate de novo lipogenesis (DNL) and hepatic triglyceride synthesis. This nutrient-specific mechanism explains why lean individuals with high sugar intake can develop MASLD at rates comparable to obese patients. Supporting evidence shows that elevated glucose induces ChREBP expression and target gene activation, thereby predisposing to steatosis [36, 37]. Kupffer cell activation further amplifies this effect, as pro-inflammatory M1 polarization enhances TNF-α production, which impairs hepatic insulin signaling and promotes lipid accumulation [38]. The combined nutrient composition of the diet, dominated by refined carbohydrates and unhealthy fats, fosters oxidative stress and inflammation, reinforcing hepatic fat deposition in lean individuals [39]. Additionally, PNPLA3 rs738409 variants, which are highly prevalent in Egypt [6], may exacerbate lipid droplet accumulation, highlighting the importance of genetic screening in difficult-to-treat cases.

Taken together, the evidence indicates that MASLD in lean Egyptians is more strongly influenced by nutrient composition, oxidative stress, and lipogenic programming than by excessive caloric intake alone. Consequently, nutritional strategies prioritizing dietary quality, such as reducing refined carbohydrates and hydrogenated fats while increasing antioxidant-rich foods, may offer more effective prevention and management than calorie reduction alone.

Over six months, body weight decreased by an average of 10.9 kg (Table 3), exceeding the 7% threshold typically associated with reversal of early-stage steatosis. Fat mass declined by 9.2 kg, while BMI was reduced by 3.9 kg/m^2^, confirming a substantive loss of adiposity rather than fluid or lean tissue. Waist-to-hip ratio improved by 0.04, consistent with reductions in visceral fat, a major contributor to hepatic lipid accumulation. On the biochemical level, ALT and AST fell by more than 30% (− 22.2 and − 21.3 U/L, respectively) (Table 4), indicating improvement in hepatocellular injury. In contrast, triglycerides showed minimal change (− 13.2 mg/dL, nonsignificant), and glucose reductions were modest. Fasting glucose declined by 8.5 mg/dL (Table 4), a statistically significant effect (*P* < 0.001) but with a smaller effect size (Cohen’s *d* = 0.6) compared to hepatic or inflammatory markers, suggesting that sustained glycemic improvement may require longer interventions or adjunct therapies.

The reduction in muscle mass observed in this study (− 3.1 kg; 9.0%) raises important considerations regarding sarcopenic risk in patients undergoing dietary therapy for MASLD (Table 3). Although some lean mass loss is physiologically expected during weight loss, particularly in individuals with high baseline adiposity (mean BMI 38.6 kg/m^2^), the degree of muscle loss seen here may reflect early, rapid weight reduction or inadequate anabolic stimulus. Our diet was protein-optimized (1.2–1.5 g/kg/day), exceeding standard dietary recommendations, and physical activity improved; however, the absence of structured resistance training likely limited muscle preservation. Future interventions may achieve better lean mass retention by integrating progressive resistance exercise, targeted amino acid supplementation, and phased caloric restriction, particularly for patients with baseline sarcopenia or severe obesity.

The dissociation between triglyceride and glucose responses is noteworthy. In Western MASLD, improved insulin sensitivity typically normalizes both parameters, yet here, triglycerides showed only modest reduction, suggesting persistent hepatic de novo lipogenesis (DNL) despite weight loss. Fructose consumption activates carbohydrate response element-binding protein alpha (ChREBPα), which upregulates lipogenic enzymes such as acetyl-CoA carboxylase 1 (ACC1) and fatty acid synthase (FAS), thereby sustaining DNL even after weight reduction [40]. The smaller improvement in fasting glucose (− 8.5 mg/dL) compared with hepatic fat reduction (− 89.5 dB/m) further implies that nutrient-specific pathways, particularly fructose-driven ChREBPα activation, can decouple glycemic control from steatosis improvement. Rodent models simulating Egyptian diets (high white rice, minimal fiber) demonstrate that ChREBPα remains transcriptionally active even after weight loss, maintaining ACC1 and FAS expression [41, 42]. This evidence supports the observation that liver fat content can improve more readily than circulating triglyceride levels during dietary interventions, highlighting the nutrient-specific effects of fructose on lipid metabolism and emphasizing the importance of dietary composition, particularly fructose restriction, beyond caloric reduction alone.

ALT and AST improvements parallel the resolution of inflammation, particularly the 53.2% reduction in TNF-α (Table 5). TNF-α promotes hepatocyte insulin resistance via JNK-1–mediated phosphorylation of IRS-1, which disrupts downstream PI3K/AKT signaling and impairs glycogen synthesis [43]. Both enzymes are released during hepatocellular injury; their decline reflects improved liver function and reduced cellular damage. The steep reduction in ALT and AST suggests restoration of insulin signaling within hepatocytes, often preceding normalization of systemic metabolic markers. This aspect is frequently overlooked in Western Mediterranean diet trials, which emphasize LDL cholesterol or HOMA-IR rather than hepatic insulin sensitivity as a primary outcome [44]. The high carbohydrate burden typical of Egyptian diets (Table 2) may prolong hepatic insulin resistance relative to European cohorts, explaining why hepatic recovery (ALT and AST decline) can precede systemic recovery (triglyceride and glucose changes lagging) [45]. These observations highlight the importance of longer interventions and adjunct strategies, such as structured physical activity or pharmacologic support, to fully correct lipid abnormalities in this population.

Smoking appeared to influence inflammatory status, with current smokers showing modestly higher baseline TNF-α levels, consistent with the pro-inflammatory effects described by Marti-Aguado et al. (2022) [46]. However, smoking status was not significantly associated with CAP score reduction, likely reflecting the small number of smokers in the cohort. These results emphasize the need for larger, stratified analyses to clarify the impact of smoking on MASLD outcomes in Egyptian populations.

The immune system is a key mediator of metabolic stress in MASLD, with the spleen contributing to systemic inflammation via IL-6, TNF-α, and chemokine production [47]. Post-intervention reductions in IL-6 and TNF-α (Table 5) indicate attenuation of immune-mediated inflammation, potentially through splenic pathways. Although spleen volume was not assessed, its recognized role in obesity- and diabetes-related complications warrants further study. Future investigations should evaluate whether hypocaloric diets influence splenic morphology in MASLD, given the implications for long-term metabolic health. The observed cytokine reductions further support the concept that dietary interventions can modulate immune–metabolic cross-talk.

After six months, CAP scores declined by an average of 89.5 dB/m, corresponding to a 29.3% reduction (Table 4), among the largest non-pharmacological improvements reported in Middle Eastern cohorts. Clinically, 60% of participants demonstrated categorical improvement in steatosis grade (Table 12), with many transitioning from severe (S3) to moderate or mild disease. In contrast, 40% showed no grade migration despite weight loss and dietary adherence, indicating heterogeneity in hepatic responsiveness. These findings highlight that caloric restriction, when culturally adapted, can substantially reduce hepatic lipid burden in most patients, though inter-individual variability remains significant.

The persistence of non-responders despite weight loss and biochemical improvement suggests a genetic contribution. The PNPLA3 rs738409 variant, particularly the I148M allele, is prevalent in North African and South Asian populations and disrupts hepatocellular lipid droplet remodeling, thereby limiting triglyceride mobilization in response to caloric restriction. Egyptian carriers of the GG genotype exhibited more severe steatosis and smaller reductions in hepatic fat despite dietary adherence [48]. Similarly, a South Indian study reported nearly 50% attenuation of liver fat improvement among carriers compared with non-carriers [49], and findings in Asian Indians confirmed that the PNPLA3 variant significantly blunted hepatic fat reduction despite comparable weight loss [50]. These results support the role of genetic screening in identifying patients who may require adjunct therapies in addition to caloric restriction.

The modest and statistically non-significant reduction in triglycerides (− 13 mg/dL, *P* = 0.514) contrasts with improvements in other metabolic parameters. This may reflect baseline dietary habits, including frequent consumption of refined carbohydrates (daily rice/macaroni: 83.3%; weekly white bread: 70%) and intermittent intake of sugar-sweetened beverages, which sustain lipogenic activity despite overall caloric restriction. Although the intervention partially modified these exposures, residual dietary effects or genetic influences on triglyceride metabolism (e.g., PNPLA3 or APOA5 polymorphisms) may have contributed. Because fructose intake and genetic markers were not quantified, this interpretation remains speculative and should be regarded as hypothesis-generating.

Responders displayed metabolic signatures consistent with mitochondrial adaptation. A strong inverse correlation between CAP change and SOD activity (*r* = − 0.65) (Table 9) indicates that reductions in hepatic fat were linked to enhanced oxidative capacity. The 209% rise in SOD activity (Table 5) is consistent with activation of PGC-1α–mediated mitochondrial biogenesis, a mechanism shown to reverse steatosis in animal models and primary hepatocytes; overexpression of PGC-1α promotes mitochondrial proliferation and fatty acid oxidation, thereby reducing hepatic lipid accumulation [51]. Dietary α-tocopherol derived from sunflower oil (Table 2) may have contributed, as this lipid-soluble antioxidant upregulates PPARγ expression and mitochondrial gene transcription in vitro. Furthermore, γ-tocopherol, another isoform of vitamin E, exerts an even stronger modulatory effect on PPARγ, with potential implications for lipid metabolism and mitochondrial function [52, 53].

One of the most striking outcomes was the 53.2% reduction in TNF-α (Table 5), which mediated 32% of the observed CAP improvement independently of BMI or weight change (Table 11). SOD activity increased more than threefold (+ 209%) (Table 5), while MDA levels declined by 41.7%. Both SOD and MDA changes were strongly correlated with CAP reduction (*r* = − 0.65 and *r* = 0.72, respectively) (Table 9). Together, TNF-α, SOD, and CAP form a biochemical triad indicating that inflammatory and redox resolution occurred in parallel, potentially triggering hepatic metabolic recovery beyond adipose loss.

Mediation analysis confirmed that TNF-α reduction explained 32% of the CAP score improvement independently of weight loss, whereas BMI-mediated effects were smaller and non-significant (Table 11). This underscores the weight-independent anti-inflammatory pathways through which dietary interventions improve hepatic outcomes in MASLD, particularly in lean phenotypes. Mechanistically, TNF-α activates the NLRP3 inflammasome in hepatocytes, leading to IL-1β release. IL-1β then upregulates SREBP-1c, enhancing lipogenic gene transcription and aggravating steatosis. Suppressing TNF-α interrupts this cascade, deactivating NLRP3 and uncoupling inflammation from lipid synthesis [54, 55].

In Egyptian patients, chronic exposure to pro-oxidants such as hydrogenated oils may preferentially activate hepatic TNF-α signaling, explaining the disproportionate benefit of inflammation resolution [52, 53]. The concurrent rise in SOD activity and decline in MDA (Table 5) suggest a marked improvement in mitochondrial redox balance, stabilizing electron transport and reducing reactive oxygen species spillover. Dietary α-tocopherol from sunflower oil (Table 2) may have contributed, as this lipid-soluble antioxidant scavenges lipid radicals and preserves mitochondrial membrane integrity. In experimental MASLD, α-tocopherol supplementation reduced hepatic MDA and increased antioxidant enzymes, including SOD, reflecting improved hepatic oxidative status [56]. Moreover, α-tocopherol enhanced expression of carnitine palmitoyltransferase-1 (CPT-1), promoting β-oxidation and reducing lipid accumulation [57].

These data indicate that hepatic fat clearance was not solely metabolic but was also driven by immunometabolic and redox improvements, highlighting therapeutic pathways often overlooked in calorie-focused interventions.​

Subgroup analysis demonstrated that lean participants (BMI < 25) experienced a greater reduction in TNF-α (− 58.2%) than obese participants (− 49.7%), despite losing significantly less weight (Table 13). This dissociation between inflammation and adiposity highlights phenotype-specific mechanisms in MASLD progression. Regression analysis identified baseline CAP score as the strongest predictor of hepatic improvement (β = 0.52), exceeding conventional markers such as BMI or HOMA-IR (Table 8). Logistic regression further indicated that lower baseline TNF-α levels and reduced intake of hydrogenated fats (Table 10) were independent predictors of achieving ≥ 10% CAP reduction. These patterns suggest that inflammatory burden may be a more relevant therapeutic target than body size in certain patient subgroups.

The lean MASLD phenotype in this cohort resembles the metabolically obese normal weight (MONW) model, characterized by visceral adiposity and hepatic lipotoxicity despite a normal BMI. A key contributor is the PNPLA3 rs738409 polymorphism, particularly the G allele, which impairs lipid droplet remodeling and promotes hepatic fat accumulation. This variant is more frequent among lean MASLD patients and has been associated with more severe histological outcomes, including steatohepatitis and fibrosis [58, 59].

Dietary patterns may further exacerbate the lean MASLD phenotype. Frequent use of hydrogenated oils and low fruit intake (Table 2) likely deplete hepatic glutathione reserves, elevate oxidative stress, and activate Kupffer cells. A recent review emphasized oxidative stress as a central driver of fatty liver disease, particularly in individuals with normal BMI [60]. However, that analysis did not identify TNF-α as a mediator of hepatic improvement, a relationship demonstrated in the mediation analysis presented here (Table 11).

hese insights carry therapeutic implications. Conventional MASLD management prioritizes weight reduction, but in lean phenotypes, targeting inflammation and oxidative stress may provide greater clinical benefit. Bioactive compounds such as curcumin, resveratrol, and tocotrienols directly inhibit NF-κB and NLRP3 signaling, attenuating hepatic inflammation without major weight loss [61, 62]. Incorporating such strategies could improve outcomes in lean MASLD and reduce dependence on calorie-centric interventions. Regional dietary practices in Egypt reinforce the need for phenotype-specific approaches beyond Western treatment paradigms.

Dietary adherence was high, with 83.6% of participants achieving ≥ 80% compliance (Table 15), and adherence correlated strongly with CAP reduction (*r* = − 0.71), making it one of the most predictive behavioral factors. Sleep quality also improved, with insomnia (AIS ≥ 6) declining from 40 to 17% (Table 7), despite the absence of a dedicated sleep intervention. These parallel improvements suggest systemic benefits of dietary modification, whereby reduced inflammatory load and improved nutrient density extended beyond hepatic outcomes to domains such as circadian regulation and neuroendocrine health. Enhanced energy, mood, and self-regulation were consistent with the biochemical improvements in inflammatory markers and sleep quality. Patient-reported barriers also contextualize the heterogeneous triglyceride response and highlight the importance of tailored behavioral support in dietary interventions.

High adherence likely reflected culturally adapted dietary modifications, particularly substituting refined starches with more nutrient-dense alternatives. Replacing white rice and bread with brown rice and whole-grain bread reduced caloric intake while providing fermentable fibers that modulate postprandial insulin and stimulate GLP-1 secretion in the distal gut. GLP-1 delays gastric emptying, suppresses appetite, and regulates mesolimbic dopamine pathways involved in reward-driven eating. Recent randomized trials confirm that whole-grain fibers enhance GLP-1 response and reduce postprandial glucose levels in healthy adults [63]. This mechanism may explain both the high adherence rates and the concurrent improvement in sleep quality.

The relationship between diet and circadian rhythm is biologically plausible. Melatonin production, which regulates sleep, depends on serotonin synthesis in the pineal gland, where the rate-limiting enzyme tryptophan hydroxylase is highly sensitive to oxidative stress. Elevated oxidative markers such as MDA (Table 5) can impair this enzyme and disrupt serotonin availability. The post-intervention reduction in MDA likely preserved pineal serotonin, enhancing melatonin synthesis and supporting improved sleep. Evidence from dietary studies in metabolic disorders shows that reducing oxidative stress with antioxidant-rich foods improves both sleep quality and circadian alignment [64]. These findings reinforce the concept that lifestyle-based MASLD therapies extend beyond hepatic outcomes to confer systemic benefits, including neuroendocrine health and circadian regulation [59].

Triglyceride levels showed only a modest, nonsignificant change (− 13 mg/dL, *P* = 0.514), contrasting with the marked improvements in steatosis, transaminases, and inflammatory markers. While earlier analysis highlighted nutrient-specific mechanisms sustaining lipogenesis despite weight loss (Table 4), additional factors likely contributed. A small non-adherent subgroup showed slight TG increases, influencing the overall effect size (Table 16). Moreover, all participants reported monthly canned juice intake, and 60% consumed carbonated soft drinks (Table 2), suggesting intermittent fructose exposure that may have sustained hepatic de novo lipogenesis [41]. The six-month duration may also have been insufficient for lipid normalization, which often requires longer interventions [14]. Finally, PNPLA3 rs738409 polymorphisms, prevalent in Egyptian populations, are associated with reduced TG responsiveness despite hepatic fat reduction [69]. Thus, the blunted TG response likely reflects the combined effects of residual fructose intake, partial non-adherence, limited intervention duration, and genetic predisposition.

**Table 16:** Triglyceride changes by dietary adherence status

Adherence Group	n	Adherence Score (mean ± SD)	Baseline TG (mg/dL)	6-Month TG (mg/dL)	ΔTG (mean ± SD)	*P*-value
Adherent (≥ 80%)	26	86.2 ± 4.7	150 ± 42	132 ± 37	− 18 ± 31	—
Non-adherent (< 80%)	4	72.5 ± 5.3	159 ± 55	164 ± 49	+ 5 ± 46	0.21

*TG* Triglycerides, *ΔTG* change from baseline; *P*-value by independent t-test

The findings of this trial carry important clinical and public health implications for MASLD management, particularly in Middle Eastern and North African populations characterized by high-carbohydrate diets and limited access to pharmacotherapy. A culturally adapted hypocaloric diet improved hepatic steatosis, reduced inflammatory burden, and enhanced antioxidant capacity, underscoring the central role of lifestyle-based interventions in reversing the metabolic and hepatic complications of MASLD. Importantly, this approach represents a scalable, low-cost strategy that could be integrated into primary care and community health programs in resource-limited settings, where MASLD prevalence is rapidly increasing.

Although Mediterranean diets demonstrate efficacy in European MASLD cohorts, their reliance on olive oil, seafood, and fresh produce may limit feasibility in low-resource Egyptian contexts. The adapted plan substituted culturally familiar foods (e.g., legumes, sunflower oil) while retaining anti-inflammatory and metabolic targets [65]. Scaling such a model within Egypt’s public health system may encounter barriers, including the cost of whole foods, limited access to clinical dietitians, and cultural preferences for carbohydrate-heavy meals. However, community-based implementation using trained lay health workers, mobile tracking tools, and simplified adherence metrics could support integration into broader MASLD prevention and treatment frameworks, particularly in under-resourced areas [27].

These findings are consistent with prior reports demonstrating the benefits of caloric restriction on hepatic and metabolic outcomes. Ghaemi et al. showed that modest weight loss through a low-calorie diet improved liver enzymes and insulin resistance in MASLD patients [67]. Likewise, Kord Varkaneh et al. reported that a 5:2 intermittent fasting regimen significantly reduced hepatic steatosis and transaminase levels, supporting the efficacy of structured dietary interventions [68]. The present data extend this evidence by demonstrating that a culturally adapted hypocaloric diet improves hepatic and inflammatory outcomes not only in obese but also in lean MASLD phenotypes, underscoring the need for phenotype-specific dietary strategies in clinical practice.

Study Strengths and Limitations.

While structured hypocaloric dietary interventions targeting hepatic steatosis and inflammatory markers are scarce in Egyptian MASLD research, existing studies provide insights into the role of lifestyle factors. For instance, one study identified significant associations between dietary habits, physical activity, sleep quality, and MASLD severity [65]. Another highlighted lifestyle contributors to MASLD risk among young Egyptian adults [27]. Additionally, a Mediterranean diet intervention demonstrated improvements in liver health among MASLD patients in Egypt [66]. Building on this limited but important body of work, our study is among the first prospective interventional trials in Egypt to comprehensively evaluate not only hepatic fat reduction but also inflammatory modulation and oxidative stress improvement following a culturally adapted hypocaloric diet. The identification of TNF-α reduction as an independent, and weight-independent, mediator of liver fat reduction provides potential associations insight into the inflammatory nature of MASLD, particularly in lean phenotypes. Furthermore, the greater hepatic and inflammatory responsiveness observed in lean MASLD patients challenges the traditional weight-centric paradigm of disease management and underscores the need for phenotype-specific dietary strategies tailored to the Egyptian population.

While the intervention showed promising clinical effects, its scalability within Egypt’s public healthcare system presents several challenges. Access to trained dietitians is limited, particularly in rural or underserved regions, and individualized dietary counseling may not be feasible in primary care settings without workforce expansion. Additionally, cost considerations may hinder adherence to certain recommended foods (e.g., lean proteins, fresh produce), particularly among lower-income groups. To address these barriers, future efforts should explore simplified dietary guidelines adapted to local food availability, group-based counseling models, and integration with existing public health infrastructure. Community health workers and mobile health platforms may also play a role in delivering education and follow-up at scale. Implementation research will be essential to determine how best to deliver such interventions in a cost-effective and sustainable manner.

Despite its strengths, this study has several limitations that warrant consideration. First, the relatively small sample size and single-center design may limit the generalizability of the findings to broader MASLD populations, particularly in diverse ethnic, cultural, or healthcare settings. Second, although the pre–post design helps control for inter-individual variability, the absence of a randomized control group limits causal inference. Without a comparator arm, it is not possible to definitively attribute observed improvements to the intervention alone, as unmeasured factors such as regression to the mean, secular trends, or concurrent behavior changes could have influenced the outcomes. This design was chosen deliberately due to feasibility and ethical concerns in a population lacking access to structured nutritional care. Nonetheless, the consistency and magnitude of improvements across multiple biomarkers (e.g., CAP, TNF-α) support biological plausibility. Third, while we performed subgroup, regression, and mediation analyses to explore potential predictors and mechanisms, these were strictly hypothesis-generating and interpreted with appropriate caution given the limited statistical power. In particular, the lean MASLD subgroup (n = 6) was underpowered. Although these patients appeared to demonstrate greater improvements in hepatic fat and inflammatory markers despite less weight loss, such findings should be considered exploratory. The small sample size precludes definitive conclusions or broad generalization to lean MASLD populations. These analyses were included to inform future hypothesis-driven studies but should not be overinterpreted. Fourth, the study duration was limited to six months, precluding evaluation of the long-term sustainability of dietary adherence and hepatic outcomes. Fifth, although patients with severe anemia (hemoglobin < 8 g/dL) were excluded for ethical and safety reasons, one-third of participants had mild-to-moderate anemia. While no adverse hematological events occurred and hemoglobin remained stable throughout the intervention, our findings cannot be generalized to individuals with more severe anemia. Sixth, the voluntary nature of enrollment may have introduced selection bias, favoring participants with higher intrinsic motivation, health literacy, or dietary interest, potentially leading to better adherence and greater observed benefit than might occur in the general MASLD population. Additionally, the lack of blinding in both participants and investigators may have contributed to detection bias, particularly in subjective outcomes like self-reported lifestyle improvements. Finally, although we tracked body composition, we did not assess muscle strength or physical performance, which would be required for a comprehensive evaluation of sarcopenia risk.

Future research should: (1) evaluate iron supplementation or micronutrient fortification in anemic subgroups; (2) validate findings in multi-center randomized controlled trials with longer follow-up (> 12 months) across diverse populations; and (3) incorporate functional sarcopenia assessments (e.g., grip strength, Short Physical Performance Battery) to fully assess musculoskeletal outcomes.

## Conclusion

A culturally tailored hypocaloric diet significantly reduced hepatic steatosis, systemic inflammation, and oxidative stress in Egyptian MASLD patients. These benefits were associated with, but not solely dependent on, weight loss. Preliminary findings in lean individuals suggest a potentially distinct response pattern, but the small subgroup size precludes definitive conclusions. Reduction in liver fat was partially explained by improvements in TNF-α levels, suggesting a possible role for inflammation resolution in MASLD recovery that merits further investigation. These findings highlight the importance of incorporating anti-inflammatory and antioxidant strategies into dietary interventions. The heterogeneity of response observed across phenotypes underscores the need for future studies to tailor interventions based on MASLD subtypes. Tailored hypocaloric diets remain a promising, low-cost first-line approach for MASLD management in resource-limited settings. The observed improvements in physical activity and sleep further support their holistic benefit. Larger randomized trials are needed to confirm these effects and clarify underlying mechanisms.

## Data Availability

All relevant data are included in this published article.
